# Estimating provisional margins of exposure for data-poor chemicals using high-throughput computational methods

**DOI:** 10.3389/fphar.2022.980747

**Published:** 2022-10-07

**Authors:** Chantel I. Nicolas, Matthew W. Linakis, Melyssa S. Minto, Kamel Mansouri, Rebecca A. Clewell, Miyoung Yoon, John F. Wambaugh, Grace Patlewicz, Patrick D. McMullen, Melvin E. Andersen, Harvey J. Clewell III

**Affiliations:** ^1^ Office of Chemical Safety and Pollution Prevention, US EPA, Washington, DC, United States; ^2^ Ramboll, Raleigh, NC, United States; ^3^ ScitoVation, Raleigh, NC, United States; ^4^ National Toxicology Program Interagency Center for the Evaluation of Alternative Toxicological Methods, Research Triangle Park, NC, United States; ^5^ 21st Century Tox Consulting Chapel Hill, Washington, NC, United States; ^6^ Center for Computational Toxicology and Exposure Office of Research and Development, US EPA, Research Triangle Park, NC, United States

**Keywords:** Threshold of toxicological concern (TTC), computational toxicology, in silico, high-throughput risk prioritization, margin of exposure (MoE)

## Abstract

Current computational technologies hold promise for prioritizing the testing of the thousands of chemicals in commerce. Here, a case study is presented demonstrating comparative risk-prioritization approaches based on the ratio of surrogate hazard and exposure data, called margins of exposure (MoEs). Exposures were estimated using a U.S. EPA’s ExpoCast predictive model (SEEM3) results and estimates of bioactivity were predicted using: 1) Oral equivalent doses (OEDs) derived from U.S. EPA’s ToxCast high-throughput screening program, together with *in vitro* to *in vivo* extrapolation and 2) thresholds of toxicological concern (TTCs) determined using a structure-based decision-tree using the Toxtree open source software. To ground-truth these computational approaches, we compared the MoEs based on predicted noncancer TTC and OED values to those derived using the traditional method of deriving points of departure from no-observed adverse effect levels (NOAELs) from *in vivo* oral exposures in rodents. TTC-based MoEs were lower than NOAEL-based MoEs for 520 out of 522 (99.6%) compounds in this smaller overlapping dataset, but were relatively well correlated with the same (*r* ^*2*^ = 0.59). TTC-based MoEs were also lower than OED-based MoEs for 590 (83.2%) of the 709 evaluated chemicals, indicating that TTCs may serve as a conservative surrogate in the absence of chemical-specific experimental data. The TTC-based MoE prioritization process was then applied to over 45,000 curated environmental chemical structures as a proof-of-concept for high-throughput prioritization using TTC-based MoEs. This study demonstrates the utility of exploiting existing computational methods at the pre-assessment phase of a tiered risk-based approach to quickly, and conservatively, prioritize thousands of untested chemicals for further study.

## Highlights

• Over 45,000 environmental chemicals have been used to generate decision-tree-derived thresholds of toxicological concern (TTCs) and predicted exposures for estimating provisional margins of exposure (MoEs).
• In vitro-derived MoEs for 709 ToxCast compounds based on oral equivalent doses (OED)s were compared with their respective TTC-based MoEs to evaluate the consistency of these alternative high-throughput risk-based prioritization metrics.
• Published *in vivo* regulatory reference values were used to demonstrate the potential to ground-truth the chemical prioritization approaches.
• The comparison demonstrates that both TTCs and OEDs provide a generally conservative surrogate for NOAELs in the absence of traditional chemical-specific experimental data.

## 1 Introduction

Chemical risk evaluation balances two fundamental concepts: hazard and exposure. The ratio of hazard to exposure, known as the margin of exposure (MoE), provides an estimate of the relative risk from a chemical exposure. MoEs have great utility if determined using well-documented measured data and can influence chemical risk assessment decisions by estimating the potential for a chemical to trigger an adverse outcome in a defined exposure scenario (Pastoor et al., 2014). For example, an MoE less than one, because of the interpretation that estimated exposures are predicted to lead to bioactivity, would indicate that a compound or use scenario might warrant additional scrutiny. MoE provides a quantitative definition of risk; however, the utility of MoEs in decision-making frameworks is limited by the lack of measured hazard and exposure data. As the number of compounds in commerce today far exceeds available resources for exposure assessment and for traditional dose-response studies that depend on expensive in-life animal testing, development of higher-throughput *in silico* methods for estimating MoEs would be beneficial.

*In lieu* of a comprehensive database of hazard and exposure information, an attractive starting point for making environmental risk-based decisions is the use of new approach methodologies (NAMs), and in particular, *in silico* tools, to predict a chemical’s potential to adversely affect health for specific exposure scenarios (Wambaugh et al., 2013, Wambaugh et al., 2014; Moreau et al., 2017). While traditional in vivo-based methods comprise the accepted approach for targeted chemical testing, decision-makers across government and industry are faced with tasks of optimizing limited resources and evaluating which chemicals, of thousands, need to undergo time and resource intensive testing protocols. The process of using high-throughput risk prioritization to identify chemicals for further testing is important to fulfill a number of regulatory programs such as the Endocrine Disruptor Screening Program (US EPA, 2015), and was highlighted by the National Research Council as a critical part of the path towards 21st century risk assessment (National Research Council, 2007; National Research Council, 2012; National Academies of Sciences Engineering and Medicine, 2017). A few recent studies have examined potential high (er) throughput, *in silico* methods for chemical priority setting and have demonstrated the validity of the technique (Paul Friedman et al., 2020; Beal et al., 2022).

Under the amended Toxic Substances Control Act (TSCA), the United States Environmental Protection Agency (EPA) has developed a plan to promote alternative testing in a framework that considers estimates for both hazard and exposure (US EPA, 2018).

The ExpoCast program, developed at the U.S. EPA’s Center for Computational Toxicology and Exposure (CCTE) (Wambaugh et al., 2013) includes high-throughput exposure (HTE) predictions. These predictions are based on an optimized set of descriptors such as predicted physicochemical properties and chemical use information, which have been used as exposure surrogates in a prioritization scheme (Nicolas et al., 2018). However, providing an appropriate estimate of hazard to weigh against exposure context is essential for estimating a compound’s potential risk to human health. In the absence of toxicity data for most commercial compounds, the ToxCast program at the CCTE has applied numerous high-throughput assays across a set of over 4,500 chemicals to determine *in vitro* bioactivity (Judson et al., 2014; Richard et al., 2016; Thomas et al., 2018). For some of these compounds high-throughput *in vitro* to *in vivo* extrapolation (HT-IVIVE) has been used to estimate an oral equivalent dose (OED), essentially converting points-of-departure from *in vitro* assays to the intake of the compound that would be required to achieve the bioactive concentration in plasma (Wambaugh et al., 2015; Wetmore, 2015). However, only a fraction of ToxCast endpoints have been converted to OEDs, because dosimetry parameters (i.e., fraction unbound, hepatic clearance) are required to accurately perform HT-IVIVE (Pearce et al., 2017). The relatively low throughput of these methods for estimating OEDs experimentally makes the extension to the larger number of chemicals in commerce problematic. Nonetheless, efforts have been made to address some of these issues by predicting dosimetry parameters from chemical structure using QSAR modeling (Pradeep et al., 2020; Dawson et al., 2021; Mansouri et al., 2021).

The RISK21 (Embry et al., 2014) tiered risk assessment approach recommends using a threshold of toxicological concern (TTC)-based approach for chemicals that lack adequate exposure data and/or NOAEL data to prioritize “data-poor” chemicals of interest. In a previous application of this approach to rapidly prioritize thousands of compounds (greater than 8000), TTCs were evaluated as potential bioactivity surrogates (Patlewicz et al., 2018). This study demonstrates that TTC-based approaches can confidently estimate bioactivity and could greatly enhance the efficiency of risk-based prioritization of “data poor” compounds. A follow up study was pursued to better evaluate the TTC assignment approach, and while the approach reaffirmed that TTC may be a useful tool in the risk-based prioritization toolbox, it also identified challenges in deconstructing the Kroes workflow to move away from relying on some of the Leadscope tools to denote functional groups (Nelms, Pradeep and Patlewicz, 2019). The basis of the TTC concept is that “safe” levels of exposure (e.g. levels that would not be expected to be a safety concern) for humans can be determined from chemical structures, even when directly applicable toxicity data are unavailable (Cramer, Ford and Hall, 1978; Kroes and Kozianowski, 2002; Hartung, 2017). This approach has been used by industry and regulatory bodies (e.g., U.S. FDA and European Commission) for compounds that are not currently regulated or that lack sufficient data for making risk assessment decisions (Leeman, Krul and Houben, 2014; Feigenbaum et al., 2015; Williams et al., 2016). In fact, the EPA has reported on their own proof-of-concept case study using a combined TTC and quantitative structure activity relationship (QSAR) approach to help prioritize chemicals based on data availability in a tiered approach (US EPA, 2021).

In this work, we have first determined whether the TTC-based MoE approach would be conservative with respect to an OED-based or NOAEL-based MoE approach and then applied the TTC-based MoE approach to prioritize more than 45,000 commercial chemicals from the Collaborative Estrogen Receptor Activity Prediction Project (CERAPP) (Kamel Mansouri et al., 2016). Applying these high-throughput tools to derive various comparative MoEs creates further opportunities to prioritize chemical spaces for higher tiers of study (Moreau et al., 2017).

## 2 Materials and methods

### 2.1 *In Silico* exposure prediction

**FIGURE 1:**
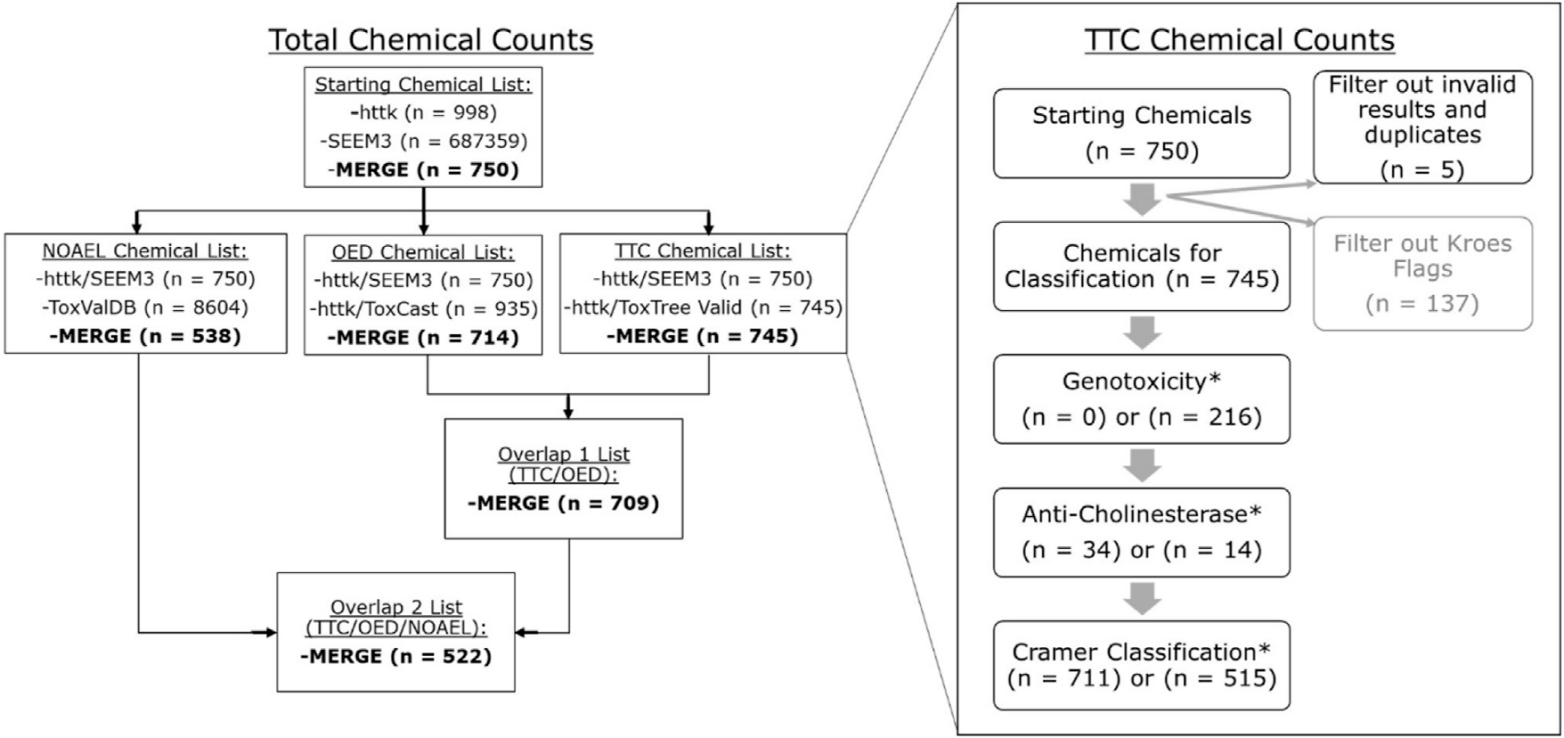
Total chemical counts and workflow for the httk dataset for both the entire study (left) and for generation of specific TTC categorizations (right). The total chemical workflow began with chemicals in both the chem. physical and *in vitro*.data list embedded in httk (*n* = 998) and the SEEM3 published values (Ring et al., 2019) (*n* > 600 k) list. Lists for TTC, OED, and NOAEL MoE calculations are separated out based on data available for the httk chemicals from a unique source (ToxTree, ToxCast, and ToxValDB, respectively). The number of chemicals in the merge between those datasets is shown in bold. Finally, the overlap between TTC and OED lists yields 709 chemicals while the overlap between TTC/OED/NOAEL lists yields 522 chemicals. The TTC workflow did not utilize the Kroes exclusion step (greyed out), which is discussed in more detail in Section 4.1. The workflow was run twice, excluding (*n* = 0) or including (*n* = 216) the genotoxicity filter.

*In vitro* metabolism data for 998 chemicals was accessed from the R “httk” package (v2.0.4) (Pearce et al., 2017) (specifically from the embedded “chem.physical_and_in_vitro.data table) and used for applying reverse dosimetry modelling. Exposures were generated using the most updated estimates from the Systematic Empirical Evaluation of Models (SEEM) calibrated consensus exposure model from U.S. EPA’s ExpoCast program (Wambaugh et al., 2013, Wambaugh et al., 2013). Estimates using SEEM3 were pulled from the Supplementary Table S1 in Ring et al., 2019 and were available for 750 of the chemicals in the small ToxCast/httk dataset (Ring et al., 2019). The overall filtering of chemicals and number of chemicals in each step for the *httk* dataset is shown in Figure 1, with subsequent sections describing the process in more detail.

### 2.2 Assignment of thresholds of toxicological concern

Various structure-based decision trees from Toxtree (v3.1.0.1851) (Patlewicz et al., 2008) were applied to the 750 chemicals from the *httk*/SEEM3 overlap dataset in addition to a larger list of approximately 45,000 chemicals (described more in Section 2.5, and also available to investigate at https://scitovation.shinyapps.io/TTCApplet/) (Nicolas et al., 2020). This approach categorized compounds into four groups: Anti-cholinesterase, Cramer Class I, Cramer Class II, and Cramer Class III. An extension here is the category: *TTC not appropriate*, identified using the Kroes TTC Decision Tree (Kroes and Kozianowski, 2002; Kroes et al., 2004) method based on the result “Risk assessment requires compound-specific toxicity data.” However, because we were looking to be more inclusive for this exploratory (i.e. not a risk assessment) analysis, we continued to include chemicals even if they were flagged in the Kroes decision tree.

As such, the 750 httk chemicals (and 45,015 CERAPP chemicals) were first run through the Structural Alerts for Functional Group Identification (ISSFUNC) (Munro, Renwick and Danielewska-Nikiel, 2008; Benigni, Tcheremenskaia and Worth, 2011) method to identify compounds that were 1) “carbamate esters” or 2) “organophosphate esters” based on a “YES” for “FG52_2” or “FG81_2”, respectively, for each chemical in the Toxtree output file. Structures that were found to have either functional group were labeled as cholinesterase inhibitors and were assigned a TTC value of 0.3 μg/kg-BW/d. The workflow was run twice, once with the usual filtering for genotoxicity and once without. The latter workflow was used for any comparisons of TTC-based MoEs with OED- or NOAEL-based MoEs. Cramer Rules (original) and Cramer Rules with Extensions (Cramer, Ford and Hall, 1978; Munro, Renwick and Danielewska-Nikiel, 2008; Patlewicz et al., 2008; Lapenna and Worth, 2011) methods were used to assign the other three classes: Low (Class I), Intermediate (Class II), and High (Class III), which correspond to 30, 9, 1.5 μg/kg-BW/d, respectively. These TTC values represent the lowest 5th percentile of the distribution of NOAELs for the compounds in the class with *in vivo* data, divided by a safety factor of 100. When filtering for the Genotoxic category was included in the workflow, compounds identified by structural alerts for genotoxic carcinogenicity or *S. typhimurium* mutagenicity were assigned a TTC value of 0.0025 μg/kg-BW/d.

The chemical structures of the HTTK dataset were retrieved *via* the U.S. EPA Comptox Chemicals Dashboard (Williams et al., 2017) using Chemical Abstracts Service Registry Numbers (CASRN). Valid TTCs were computed for 745 httk chemicals.

### 2.3 Chemicals with *in.vitro* oral equivalent doses

The ToxCast *in vitro* database (v. 3.4^a^) includes data from thousands of chemicals that underwent ToxCast high-throughput *in vitro* assay screening and was used to obtain half-maximal activity concentration (AC50) values for chemicals in the *httk* dataset. Using “httkpop” functionality within the *httk* package, human oral equivalent doses (OEDs) in mg/kg-BW/d were derived using the 5th percentile of the distribution of AC50 values, based on the estimated 95th percentile steady-state concentration (C_ss_) for each chemical. Using these criteria, OEDs were derived for 714 chemicals based on the 5th percentile distribution of their respective ToxCast AC50 values.

### 2.4 Chemicals with in vivo reference doses and No adverse effect levels

U.S. EPA’s ToxVal Database (ToxValDB v. 9.1.1^b^) was used to identify chemicals that have NOAELs based on oral exposures corresponding to various studies performed on laboratory animals. The pertinent NOAEL values were generated by first filtering ToxValDB according to the following: the “type” was filtered to only “NOAEL”, the “units” were filtered to “mg/kg-day”, and the exposure route was filtered to “oral”. From there, the NOAEL estimates were calculated by taking the 5th percentile of the NOAEL values available (or lowest available value if <20 NOAEL values are provided) for a given chemical and assuming a discontinuous function with averaging between discontinuities (type 2 in R quantile () function as in Paul Friedman et al., 2020) (Paul Friedman et al., 2020). NOAELs were available for 538 of the *httk* chemicals.

### 2.5 Selection of large chemical dataset

We enlisted a structural database of 45,015 compounds that had been featured in the Collaborative Estrogen Receptor Activity Prediction Project (CERAPP) (Kamel Mansouri et al., 2016a). During the CERAPP project, this dataset underwent a rigorous curation process using an automated workflow^c^ to standardize the chemical structures in preparation for quantitative structure activity relationships (QSARs) modeling. This workflow performs a series of operations on chemical structures including desalting, standardizing tautomers and nitro groups, correcting valence, neutralizing when possible, and removing duplicates resulting in standardized forms called “QSAR-ready” that are used on the U.S. EPA Comptox Chemicals Dashboard among other applications (K. Mansouri et al., 2016; Mansouri et al., 2018). The QSAR-ready structures from the chemical dataset are provided in terms of simplified molecular-input line-entry system (SMILES) (Weininger, 1988) strings in a. csv file. TTC values were available for all 45,015 of the chemicals (referred to as the large dataset) (Nicolas et al., 2020). ExpoCast SEEM3 upper 95% exposure estimates were available for 27,116 chemicals in the large dataset. Of the remaining 17,899 chemicals, 10,064 were not run in SEEM3. An additional 7,835 had been examined with SEEM3 and were determined to be outside the domain of applicability due to inability to assign an exposure pathway that was similar to the chemical training set (pesticidal, consumer product, dietary, and industrial chemicals) (Ring et al., 2019). Predictions from the less accurate model SEEM2 were available for all 17,899 chemicals not covered by SEEM3 and were higher (more conservative) than the SEEM3 median estimates for the 7,835 chemicals that had estimates. As such, the exposure values for these chemicals were pulled from SEEM2 upper 95% estimates.

### 2.6 Application of margins of exposures (MoEs)

Margins of exposure were calculated using the general equation:

$$MoE=\frac{PoD}{{SEEM}_{95}}$$

Where PoD is one of the three point of departure estimates, either *in vitro* OEDs derived from the 5th percentile ToxCast AC50 value (for OED-MoE), TTC value (for TTC-MoE), or NOAEL value (for NOAEL-MoE), and SEEM_95_ is the upper 95% confidence bound for the median ExpoCast SEEM exposure estimate. OED, NOAEL, TTC, ExpoCast SEEM, and MoE values for chemicals in the small (HTTK.Toxtree) and large (CERAPP.Toxtree) datasets are provided in Supporting Information.

All analysis was done with R software (v 4.2.1) integrated with RStudio (build 2022.07.1). An R markdown (rmd) file has been provided in the Supplementary Materials (along with the supporting documents) that can be run to replicate the analysis and figures escribed here. Figures and MoE outputs can be found in the “Risk_Prioritization” sub-folder.

## 3 Results

### 3.1 Comparisons of chemical prioritization metrics

**FIGURE 2:**
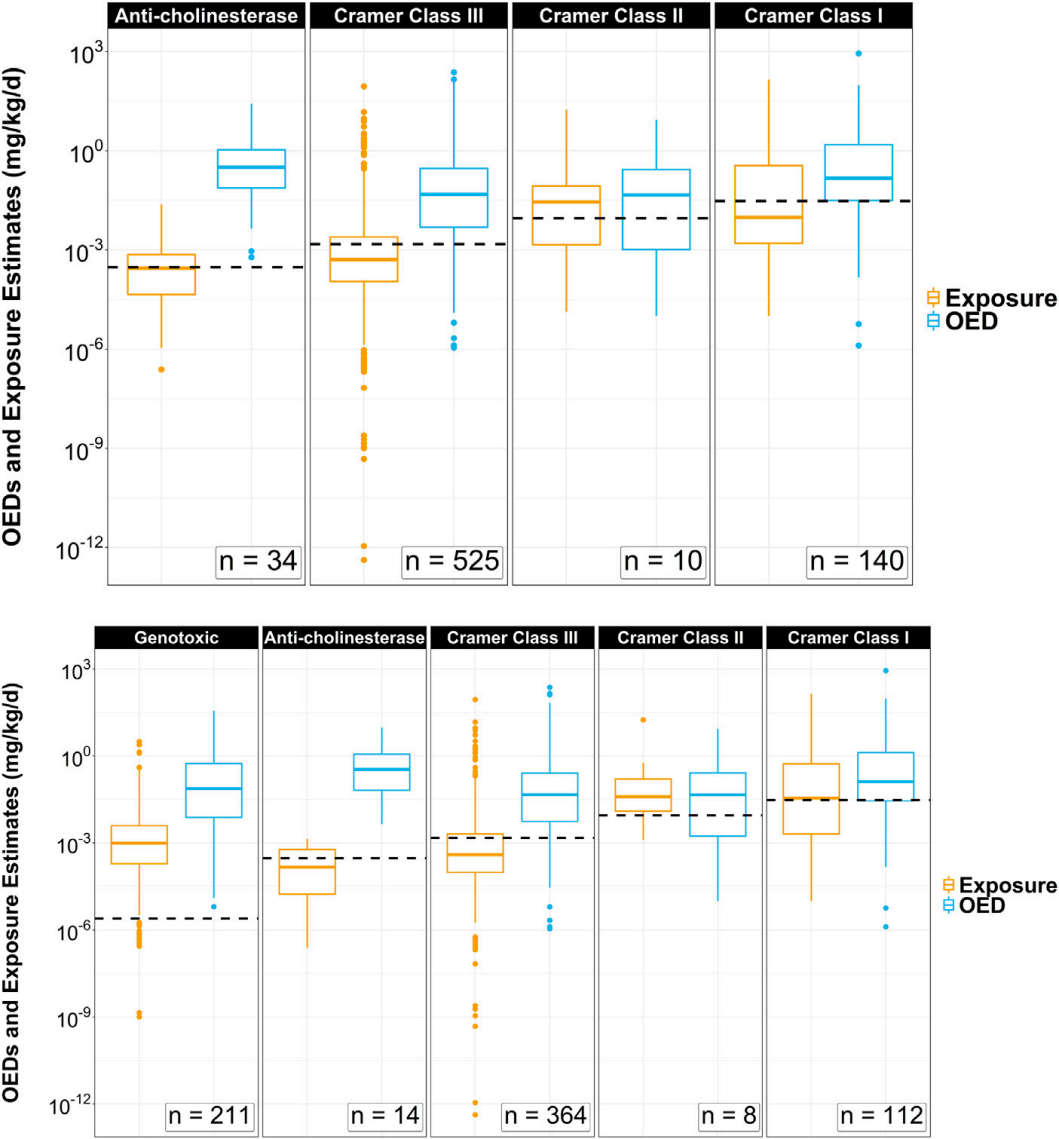
Side-by-side box and whiskers comparisons of oral equivalent doses (OEDs) and ExpoCast SEEM exposures across TTC category thresholds for 709 ToxCast chemicals that have metabolism data to support HT-IVIVE. The analysis was conducted without (top) and with (bottom) filtering for Genotoxicity. Top: The category TTC values represented by the horizontal dotted lines are 0.0025 (bottom only), 0.3, 1.5, 9, and 30 μg/kg/d. Dots above each box and whisker plot signify chemical outliers for a given range of OED or ExpoCast SEEM exposure estimates in mg/kg-BW/d.

TTC values were assigned for all 709 ToxCast chemicals in the small dataset. A total of 18.3% (*n* = 130) of the chemicals evaluated using the Kroes Decision Tree method were deemed inappropriate for Cramer classifications. These chemicals were nonetheless carried forward in the investigation. The distribution of OED values across each TTC category was plotted side-by-side with ExpoCast SEEM exposure estimates (Figure 2) with the TTC standard values for each chemical category plotted and represented by the dotted black horizontal lines. The panels are arranged such that TTCs increase from left to right (Genotoxic < Anti-cholinesterase < Cramer Class III < Cramer Class II < Cramer Class I). TTCs were generally lower than ToxCast OEDs. The number of chemicals in each of the four noncancer TTC categories is summarized in Table 1. There were significant differences between medians (Wilcoxon rank sum test) and distributions (Two-sample Kolmogorov-Smirnov test) for exposures and OEDs for chemicals categorized as Genotoxic, Anti-cholinesterase, Cramer Class III, and Cramer Class I (*p* < 0.01), but no apparent differences between those measures for Cramer Class II (*p* > 0.1).

**TABLE 1:** Summary of 709 ToxCast chemicals with computed oral equivalent doses (OEDs) in the small dataset that are classified into four noncancer TTC categories based on Cramer decision tree results, along with the number of chemicals with estimated OED- and noncancer TTC-based margins of exposure (MoEs) of less than one.

TTC class and MoE results	Number of compounds (% of compounds)	OED-based MoEs <1 (% of compounds in class)	TTC-based MoEs <1 (% of compounds in class)
Anti-cholinesterase Inhibitors	34 (4.8%)	1 (2.9%)	16 (47.1%)
Cramer Class III	525 (74.0%)	69 (13.1%)	168 (32.0%)
Cramer Class II	10 (1.4%)	4 (40.0%)	6 (60.0%)
Cramer Class I	140 (19.7%)	39 (27.9%)	60 (42.9%)
Total	709 (100%)	113 (15.9%)	250 (35.3%)

The ratio of ToxCast OEDs to ExpoCast SEEM exposure predictions, yielded an OED-based MoE of less than 1 for 15.9% of the 709 compounds (*n* = 113) (Table 1; Supporting Material). TTC-based MoEs, calculated by dividing TTC-estimates by ExpoCast SEEM exposure estimates, yielded MoEs of less than 1 for 35.3% of the compounds (*n* = 250). Cramer Class II yielded the lowest median OED-based MoE at 5.6, while anti-cholinesterase yielded the highest with 2297.5. Median TTC-based MoEs for chemicals in the order: Genotoxic (0.00255) < Cramer Class II (0.229) < Cramer Class I (0.857) < Anti-cholinesterase (2.28) < Cramer Class III (3.81). The overall lower values for noncancer TTC-based MoEs compared to OED-based MoEs are driven by the inherent conservativeness of the TTC values, particularly for chemicals with flags for anti-cholinesterase activity, which is lower than the TTC value for the most potent Cramer class (Class III) by a factor of 5.

As a proof of concept for comparing the two prioritization schemes in terms of chemical ranking, the top 25 ranked chemicals were compiled for the small dataset based on the smallest OED- and noncancer TTC-based MoE values (Table 2). All four noncancer TTC categories were represented in the top 25 chemicals ranked by both OED-based MoEs and TTC-based MoEs. Ten of the chemicals overlapped between the two sets of data indicating some overlap between prioritization using either method.

**TABLE 2:** Summary of top 25 chemicals for the small dataset ranked by increasing value of estimated margins of exposure (MoEs). OEDs were divided by ExpoCast SEEM exposure estimates to obtain OED-based MoEs. Noncancer TTCs were divided by ExpoCast SEEM exposure estimates to obtain TTC-based MoEs. Chemicals in both lists have been bolded and underlined.

OED-based		Noncancer TTC-Based	
Ranking	Chemical	Ranking	Chemical
1	**Saccharin**	1	**Nitrapyrin**
**2**	Octane	**2**	**Caprolactam**
**3**	Coumarin	**3**	**2-Mercaptobenzimidazole**
**4**	**Sodium 2-mercaptobenzothiolate**	**4**	Oleyl sarcosine
**5**	Diallyl phthalate	**5**	Bis(2-ethylhexyl) nonanedioate
**6**	**Dibutyl adipate**	**6**	Acrylonitrile
**7**	2,6-Di-tert-butyl-4-[(dimethylamino)methyl]phenol	**7**	Cyazofamid
**8**	**Nicotinic acid**	**8**	1-Benzylquinolinium chloride
**9**	**Methanol**	**9**	1,2-Dichloroethane
**10**	Estrone	**10**	**Nicotinic acid**
**11**	**Caprolactam**	**11**	3-Isopropylphenol
**12**	**1,2-Dimethyl-3-nitrobenzene**	**12**	Quinoline
**13**	**Nitrapyrin**	**13**	**Methanol**
**14**	**2,2′-Methylenebis** ( **ethyl-6-tert-butylphenol** )	**14**	1-Dodecyl-2-pyrrolidinone
**15**	2-Phenoxyethanol	**15**	Pentachloropyridine
**16**	Octanoic acid	**16**	**Dibutyl adipate**
**17**	meso-Hexestrol	**17**	**2,2′-Methylenebis** ( **ethyl-6-tert-butylphenol** )
**18**	Di (2-ethylhexyl) phthalate	**18**	**Sodium 2-mercaptobenzothiolate**
**19**	Allura Red C.I.16035	**19**	Methenamine
**20**	Dinoseb	**20**	Glycidyl trimethylammonium chloride
**21**	Decane	**21**	**Saccharin**
**22**	**2-Mercaptobenzimidazole**	**22**	4-Morpholinecarboxaldehyde
**23**	Sulisobenzone	**23**	4-Cumylphenol
**24**	Perfluorohexanoic acid	**24**	FD&C Yellow 6
25	Triphenylphosphine oxide	**25**	**1,2-Dimethyl-3-nitrobenzene**

### 3.2 Prioritization of 45000+ chemicals in commerce

Despite the many thousands of chemicals that are currently in commerce in the United States, sufficient *in vitro* metabolism data exists to estimate oral equivalent doses for only a few hundred compounds. To inform prioritization of these data-poor compounds, we determined TTC values for the chemicals in the CERAPP dataset (45,015), of which 677 were also in the smaller dataset of chemicals with ToxCast/Tox21-based OEDs. There was an overlap of only 1 chemical (nitrapyrin) between the top 25 ranking chemicals in small dataset and the top 25 ranked chemicals in the large dataset based on TTC.

Due to limitations in the structures considered by the model, the Kroes Decision Tree criteria indicated that TTC estimates were considered inappropriate for use with 5,165 (11.5%) chemicals, comparable with the smaller dataset. Like the analysis presented above, these chemicals were carried forward in this analysis. The breakdown of chemicals into the various TTC classes were 1506 (3.3%), 35,772 (79.5%), 1346 (3.0%), and 6391 (14.2%) respectively, in the Anti-cholinesterase, and Cramer Classes III, II, and I (Table 3). TTC-based MoEs of less than one were calculated for 9.4% (*n* = 4216) compounds.

**TABLE 3:** Summary of the large dataset of CERAPP chemicals that are classified into four TTC categories based on Cramer decision tree results along with the number of chemicals with estimated TTC-based margins of exposure (MoE) of less than one.

TTC class and MoE results	Number of compounds (% of compounds)	OED-based MoEs <1 (% of compounds in class)	TTC-based MoEs <1 (% of compounds in class)
Anti-cholinesterases	1506 (3.3%)	1 (0.1%)	207 (0.1%)
Cramer Class III	35,772 (79.5%)	84 (0.2%)	3,158 (8.8%)
Cramer Class II	1346 (3.0%)	6 (0.4%)	120 (8.9%)
Cramer Class I	6391 (14.2%)	23 (0.4%)	731 (11.4%)
Total	45,015 (100%)	114 (0.3%)	4216 (9.4%)

**FIGURE 3:**
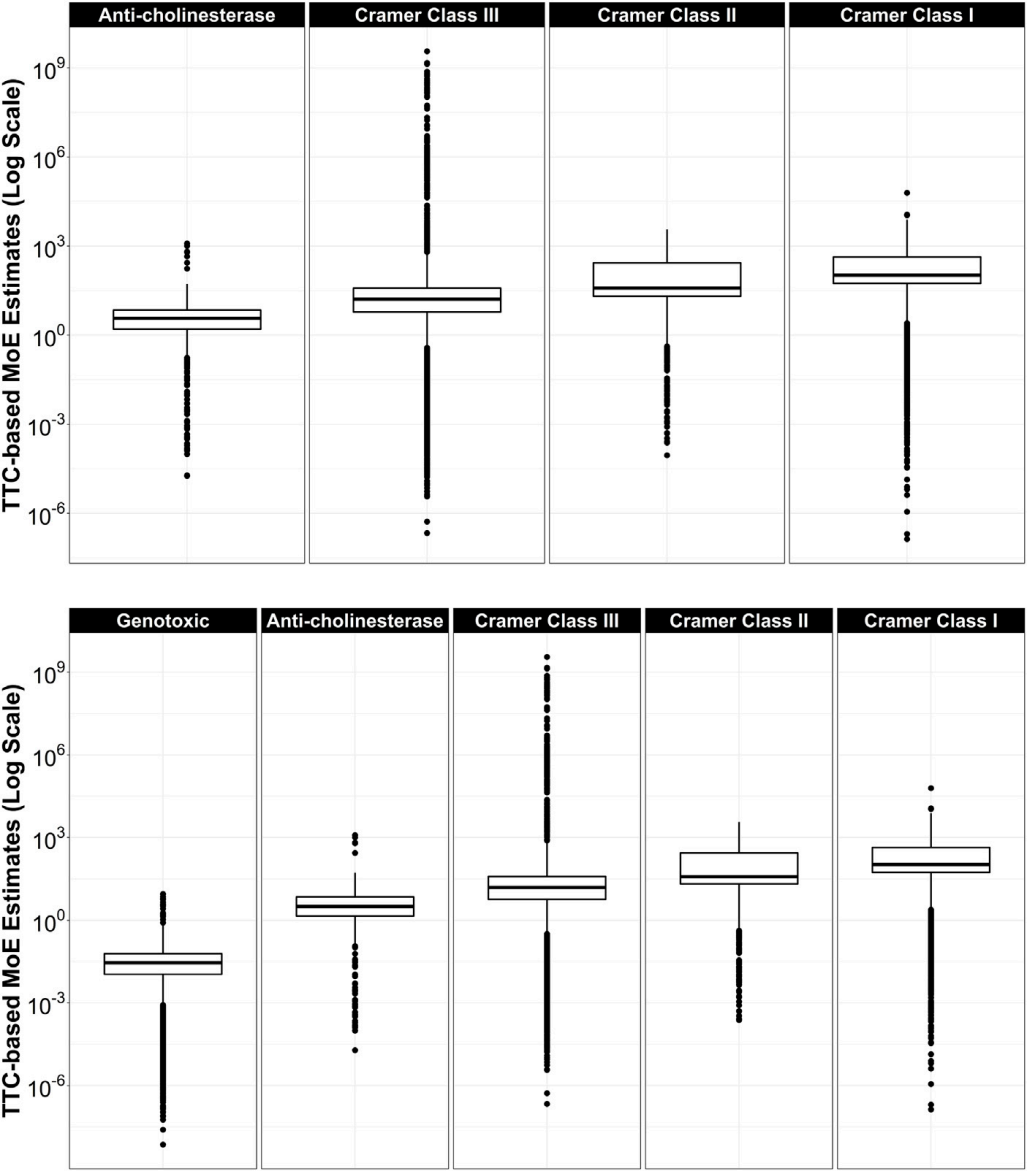
Top: TTC-based Margins of Exposure by TTC category. From left to right: Anti-cholinesterase (*n* = 1506), Cramer Class III (*n* = 35,772), Cramer Class II (*n* = 1346), Cramer Class I (*n* = 6391), for compounds that have both TTC and ExpoCast SEEM exposure estimates. Dots above each box and whisker represent chemicals with MoEs outside the 5th and 95th percent of the range. Bottom: Same as top, but with Genotoxic (*n* = 11,407) category included.

For the large dataset of CERAPP chemicals, the distribution of TTC-based MoEs within each TTC category was plotted side-by-side for all categories (Figure 3). Median TTC-based MoEs increased from left to right and varied in orders of magnitude: Genotoxic (0.0289) < Anti-cholinesterase (3.17) < Cramer Class III (15.5) < Cramer Class II (38.2) < Cramer Class I (106).

### 3.3 Ground-truthing NAM-based chemical risk prioritization using traditional *in vivo* data

**FIGURE 4:**
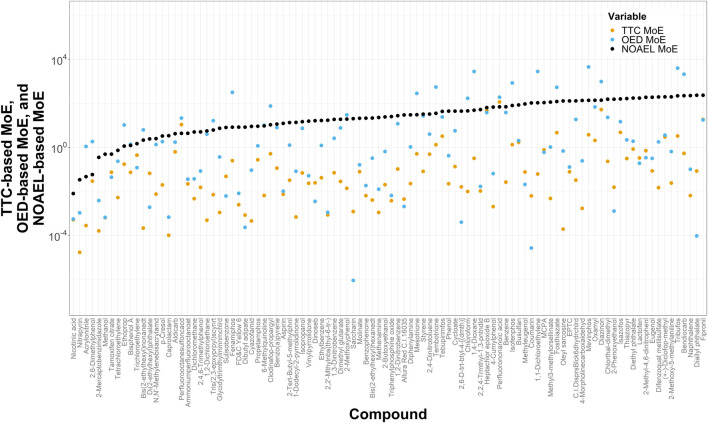
Plot of margins of exposure estimated by dividing TTCs, OEDs, and NOAELs, by ExpoCast SEEM exposures yielding TTC-, OED-, and NOAEL-based MoEs, respectively. This plot shows the lowest 100 NOAEL-based MoEs (from the dataset of 522) and shows those MoEs in increasing order from left to right. For this analysis, the filter for Genotoxicity was not applied for determining the TTC value, because the OEDs and NOAELs represent non-cancer endpoints.

To anchor our computationally derived noncancer TTC-MoEs against more conventional estimates, we compared them with MoEs derived using OEDs developed in the ToxCast program and with MoEs derived from NOAELs from ToxValDB. If a TTC value was lower than either of the respective OED or NOAEL values, then it was designated as more protective (conservative) at the pre-assessment phase. Of the 709 chemicals in the small dataset that have corresponding TTC and OED values, TTCs were more protective for 83.2% (*n* = 590) of them. Figure 4 demonstrates the lowest 100 NOAEL-based MoEs from 522 chemicals that have MoE values based on NOAELs, OEDs and TTCs, with increasing NOAEL-based MoEs from left to right. Of the 522 chemicals with all 3 measures, TTC-based MoEs were lower than OED-based MoEs for 84.1% (*n* = 439) of them.

**FIGURE 5:**
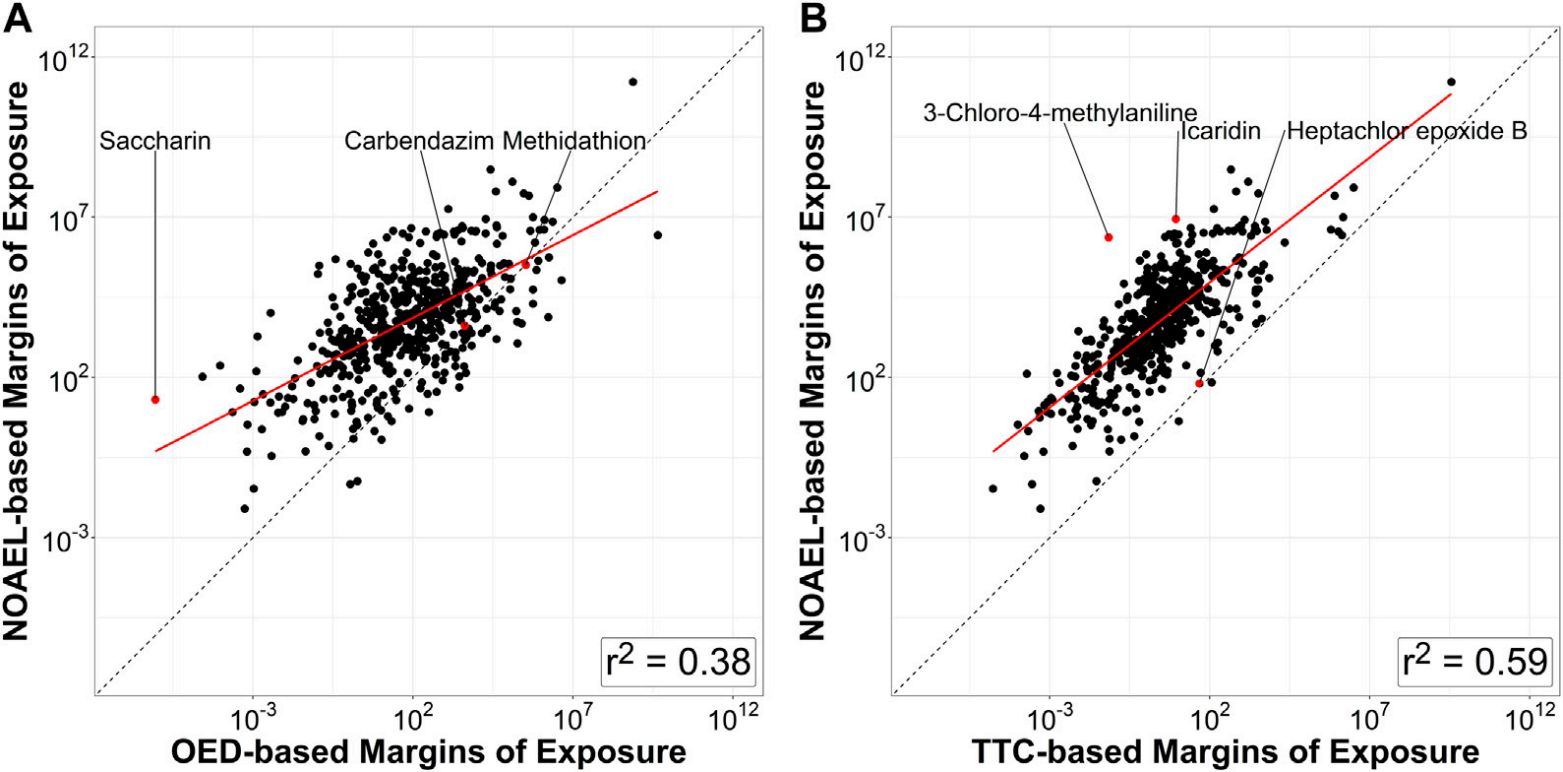
Relationship between various margins of exposure based on various estimates of chemical hazard for the 522 compounds that have all three values (NOAEL, OED, and TTC). **(A)** Margins of exposure derived from *in vivo* NOAEL values are compared to margins of exposure derived from OEDs based on ToxCast. Grey dashed line represents equivalence between the two metrics. **(B)** Margins of exposure derived from *in vivo* NOAEL values are compared to TTCs. For this analysis the filter for Genotoxicity was not applied in determining the TTC value, because the OEDs and NOAELs are based on non-cancer endpoints. All but two compounds (perfluorooctanesulfonic acid and perfluorononanoic acid) have a NOAEL-based margin of exposure that is higher than the TTC-based margin of exposure. Coefficient of determination between log_10_-transformed NOAEL-based MoE and OED-based MoE for these compounds is (*r* ^2^ = 0.38), whereas coefficient of determination between log_10_-transformed NOAEL-based MoE and TTC-based MoE is (*r* ^2^ = 0.59). Labeled points provide examples of the chemicals with the most divergent (saccharin, 3-chloro-4-methylaniline, icaridin) and similar (carbendazim, methidathion, hepatochlor epoxide B) MoE ratios.

Figure 5 shows a scatter plot of 522 NOAEL-based MoEs vs. OED-based MoEs and one of NOAEL-based MoEs vs. TTC-based MoEs. Chemicals on or close to the unity line (dotted) show good agreement between the different MoEs. Of these 522 chemicals, OED-MoEs were lower than NOAEL-MoEs for 87.5% (*n* = 457) and TTC-MoEs were lower than NOAEL-based MoEs for 99.6% (*n* = 520). Values of the NOAEL-based MoEs and OED-based MoEs were most similar for carbendazim and methidathion, while the greatest similarity in values of NOAEL- and TTC-MoEs was seen for hepatochlor epoxide B. The median of the ratio of NOAEL-based MoEs to OED-based MoEs was about 97.4 while the median of the ratio of NOAEL-based MoEs to TTC-based MoEs was 2000.

**FIGURE 6:**
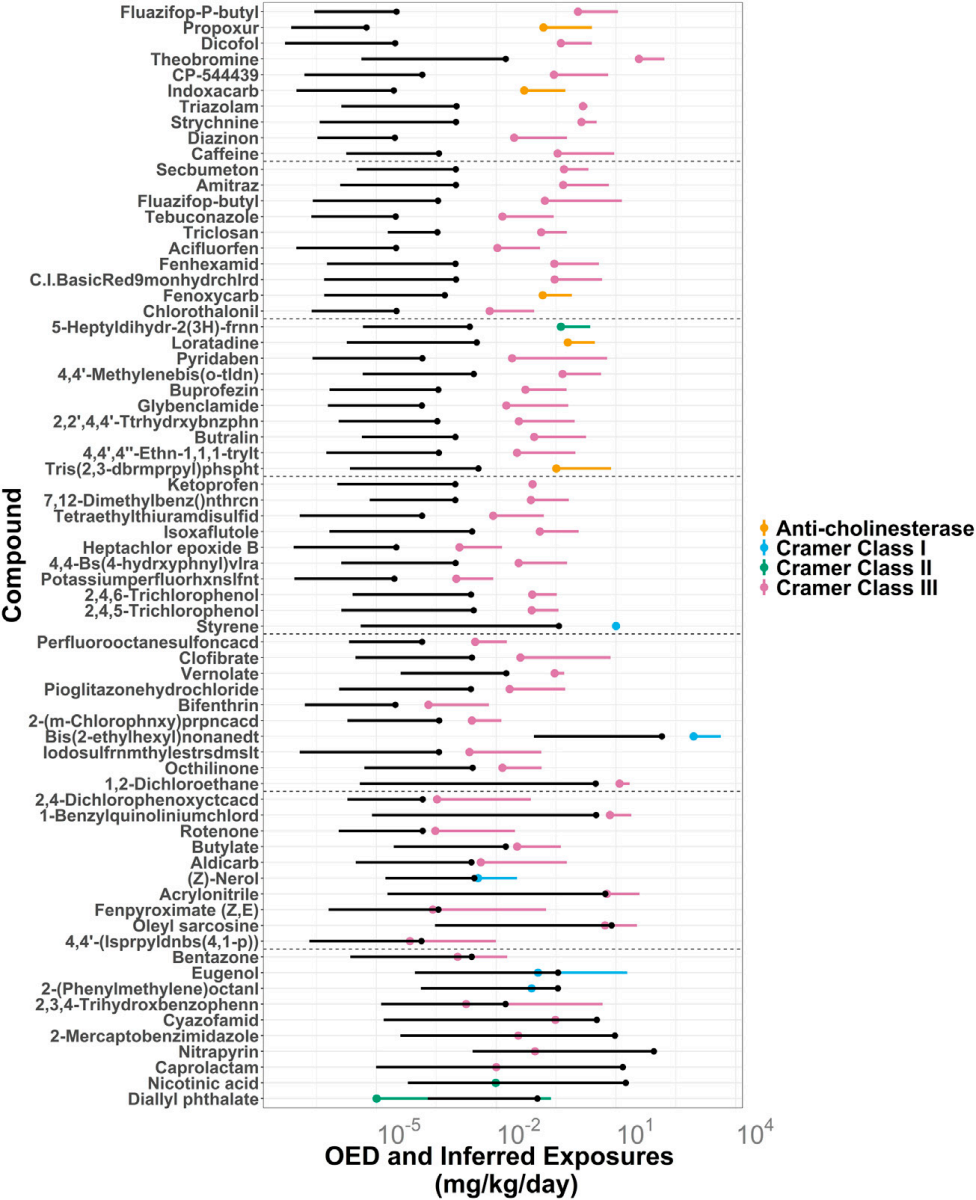
ExpoCast SEEM3 exposure estimates in black vs. oral equivalent doses (OEDs) for 70 ToxCast compounds with HT-IVIVE data grouped by the non-cancer TTC categories: Anti-cholinesterase, Cramer Class III, Cramer Class II, and Cramer Class I. The lower end of the OEDs are dots representing the OED derived from the 5th percentile AC50 values while the adjoining line segment extends up to the OED derived from the median AC50. The upper end of the exposures are black dots that represent the 95% confidence interval for the median exposure and the lower limit of the black line segments represent the median exposure estimates. The ratio of 5th percentile OED to the 95% CI for median exposure increases from bottom to top. The first 10 chemicals from each set of 100 are displayed, with dotted lines separating each set such that the first chemicals are 1–10 (the lowest ratio), the next set are 101–110, etc. For this analysis, the filter for Genotoxicity was not applied for determining the TTC value, because the OEDs are not based genotoxicity or cancer endpoints.

To further illustrate the difference between TTC-based and OED-based prioritization of the HTTK dataset, 70 chemicals with a wide range of OED-based MoEs were plotted by 5th percentile OED estimates, attached to their median OED estimates (Figure 6). The OED ranges were colored by their corresponding TTC categories: Anti-cholinesterase, Cramer Class III, Cramer Class II, and Cramer Class I. Also plotted were corresponding ExpoCast SEEM exposure ranges from median to upper 95th percentile confidence interval. Compounds with overlapping OED and ExpoCast SEEM ranges, such as diallyl phthalate, would be prioritized higher than those with higher MoEs.

## 4 Discussion

Traditional methods for chemical safety assessment (i.e. *in vivo* animal studies) are not efficient for meeting risk assessment demands in a timely manner. Thus, a tiered paradigm becomes attractive because it allows for the formal integration of emerging NAMs with traditional toxicity testing and fosters a transparent prioritization process. Even use of limited available data and computational tools could provide methods to prioritize compounds requiring the most immediate attention based on pre-assessment. While the use of *in silico* methods for estimating hazard and exposure values introduces uncertainty, they should enable industries to evaluate whether particular chemicals in development would have the potential to lead to exposures with some level of risk. However, case studies are needed to demonstrate how high-throughput risk prioritization methods can be combined with tools to estimate end-point specific toxicity and incorporate available data for structurally similar compounds (i.e., QSAR, read-across). Furthermore, success in these prioritization efforts also requires high-throughput exposure estimation tools that account for use-specific metrics (i.e., near-field use, chemical emissivity, chemical life-cycle).

### 4.1 Typical thresholds of toxicological concern process

The typical procedure for estimating the TTC involves 4 steps: Kroes decision tree inclusion, genotoxicity assessment, anti-cholinesterase identification, and finally, Cramer class determination (Kroes et al., 2004; Nelms, Pradeep and Patlewicz, 2019). In the first step, the Kroes TTC decision tree would be used to filter out chemicals that either did not fall into the chemical domain space (e.g., organo metallics, dioxin) or where daily exposures are estimated to be high enough to indicate bioaccumulation, rendering TTC less relevant in cases of lifetime exposure. From there, the chemicals would be run through the carcinogenicity and *in vitro* mutagenicity (Ames test) decision trees to determine genotoxic potential. Finally, the structural alert for functional group identification and Cramer rules (with extension) trees would be used, as in this investigation, to identify anti-cholinesterase and Cramer class I-III chemicals.

The first step (Kroes TTC decision tree) was run for this investigation and identified 57 of the 709 chemicals as being outside the typical chemical domain from TTC and an additional 80 chemicals that need additional information when daily intake values (SEEM3 median estimates) were provided. While these chemicals would typically be removed from further analysis, we decided to include them as the purpose of this investigation was exploratory, and they did not appear to have any outsized effect on the end results (e.g., exclusion of Kroes chemicals slightly improves *r* ^2^ value in Figure 5B from 0.59 to 0.61).

The second step (genotoxicity assessment) was also performed and used for some aspects of this analysis. The rationale for exclusion from comparisons with OED- and NOAEL-based MoEs was that the recommended TTC value for genotoxic compounds (216 chemicals of 709 which were classified as such), is exceptionally low (0.0025 μg/kg BW/day) even compared to anti-cholinesterase compounds TTC (0.3 μg/kg BW/day), based on an assumption of low-dose linearity for carcinogenicity. The result of using this very conservative TTC value was that a greater portion of the chemicals appeared to have TTC-MoEs << 1 and this skewed the comparison/correlation with OED-MoE and NOAEL-MoE values, which are based on noncancer effects. Although screening for carcinogenicity is an important part of risk prioritization, it is not necessarily expected that the methodologies developed for non-cancer effects should be directly applicable to cancer assessment (Thomas et al., 2013). *In vitro* and *in silico* methods have traditionally been used qualitatively to identify potential genotoxins, but quantitative assessment of genotoxicity and cancer continues to rely on *in vivo* studies. Quantitative applications have been limited by assay sensitivity, low-throughput, and lack of metabolism in the *in vitro* systems. Efforts to compare first generation ToxCast/Tox 21 data to *in vivo* cancer results demonstrated low predictivity, presumably due to the aforementioned limitations of the available assays, as well as the limited concentrations used in ToxCast (<100 µM) (Knight et al., 2009; Kligerman et al., 2015). A number of efforts are ongoing to develop and validate sufficiently sensitive, and reliable quantitative *in vitro* methods for genotoxicity endpoints e.g., (Clewell, Thompson and Clewell, 2019; Wheeldon et al., 2020; Buick et al., 2021), and to add this data to the chemistry dashboard (Hershman et al., 2017). Here, we focused on non-cancer endpoints where data is available for large numbers of chemicals. Extensions of this work to specifically focus on carcinogens are recommended as the data becomes available, however due to the complexity of the mode of action for cancer, and the widely-held assumptions embedded in the risk assessment process for carcinogens (i.e., low-dose linearity, 1 in a million lifetime acceptable risk), comparison of *in vitro* PoDs to *in vivo* cancer risk may not be as straightforward as those used for non-cancer risk assessment.

Another point of interest is the need for filtering chemicals in large databases that may not be appropriate for TTC-MoE based analysis/prioritization. One particular chemical class that may be included in these databases are volatile chemicals, which may not be valid to run through Cramer Classification, which only accounts for oral exposures, and are excluded from HTS assays. In these large databases, chemicals could be filtered out by using boiling points (e.g., OPERA-predicted boiling points <260°C) and/or vapor pressures (e.g., > California Air Resources Board cutoff, > 0.1 mmHg), indicative of a volatile chemical (US EPA, 2014; CARB, 2021).

### 4.2 Advances in comparative chemical risk prioritization metrics

OED-based MoEs require collection of basic *in vitro* pharmacokinetic (PK) parameters, including estimates of metabolic clearance. However, very limited *in vitro* metabolism data are available for most chemicals that need to be prioritized. In contrast, TTC-based MoEs can be applied to a larger number of compounds, could be used to prioritize across a greater chemical space, and for this subset of chemicals, appears to correlate better with *in vivo* NOAEL data as shown in Figure 5B. Our results diverge somewhat from a previous study that applied a TTC-based MoE approach to over 8,000 chemicals with exposure ranges consistent with biomonitoring data from the National Health and Nutrition Examination Survey (NHANES) (Patlewicz et al., 2018). In this earlier study, 47% of compounds were classified into TTC Cramer Class III compared to 79% in the CERAPP data set from our work. The majority of this discrepancy however, is due to the former study’s use of the genotoxicity categorization. When that category is used for this study, only 57% of the CERAPP chemicals are classified as TTC Cramer Class III, which is better aligned with the Patlewicz et al. results.

A variety of risk prioritization tools could be used in tandem and provide opportunities for triaging chemicals for further testing. In this study, chemical rankings varied between in vitro-derived OED-based MoEs and TTC-based MoEs, demonstrating that there may be chemical space bias even with the relatively small 709-chemical dataset.

### 4.3 Applications of thresholds of toxicological concern-based MoE as a conservative screening level approach

Comparing TTC-based MoEs and OED-based MoEs provided opportunities to explore the chemical space in which TTCs provide lower MoEs than OEDs, an outcome that may lead to the prioritization of efforts to collect reverse toxicokinetic data. To the extent that TTCs provided lower MoEs than either OEDs or NOAELs, they can be regarded as a more conservative approach to high-throughput screening assessment that correlate well (*r* ^2^ = 0.59 for 522 chemicals) with NOAEL-based MoEs. Furthermore, TTC-based MoEs appear useful as a conservative high-throughput approach for pre-assessment level risk prioritization especially because TTCs are more protective than NOAELs for 520 out of 522 chemicals. This observation further supports TTCs as a useful approach for evaluating the limited landscape of regulatory data. It should be noted that use of NOAELs to determine a human-relevant point of departure generally requires extensive curation and is a much more extensive undertaking than the scope of this study. However, for this study, we had a different focus–using the most conservative NOAEL value (or the 5% ile if > 20 NOAELs were available). As a result, we were able to compare TTC-based MoEs to NOAELs that are most likely to be protective, and it was found that the TTC-based approach was even more protective. The use of the TTC-based MoE metric as an alternative approach to looking across large numbers of compounds creates opportunities for additional read-across case studies that are based on both prioritized chemical and biological spaces (McMullen et al., 2018).

The value of applying structure-dependent TTC-based MoEs was demonstrated as a pre-assessment chemical prioritization approach to over 45,000 compounds. A slight limitation of this study was that 17,899 of the ∼45,000 chemicals investigated in our large dataset did not have SEEM3 95% upper CI estimates readily available and so SEEM2 estimates were substituted for those chemicals. While lack of the updated SEEM3 values for those chemicals may somewhat affect the outcomes for whether the MoEs are <1, the SEEM2 upper 95%ile values provide the next best estimates. Additionally, so long as all 3 MoE estimation methods (NOAEL-, OED- and TTC-based) are being normalized by the same exposure value for a given chemical, we would not expect a significant influence on the overall comparison of which method was more conservative for that chemical.

Chemical space analysis may be useful in assisting prioritization by evaluating chemical features that lead to larger TTC-based MoEs relative to those determined from lower-throughput methods. The comparison of TTC- to OED-based MoEs, where available, provided cases where the most protective value could be considered when determining which chemicals should undergo further study. Furthermore, lower-throughput exposure tools based on real-world usage patterns or exposure information could then be utilized with the high priority chemicals. The use of NOAEL-based MoEs was shown to be a potential ground-truth mechanism for TTC-based MoEs and called for both chemical and biological spaces to be prioritized for read-across case studies.

## Data Availability

The original contributions presented in the study are included in the article/Supplementary Material, further inquiries can be directed to the corresponding authors.
