# Supplementary material for: Estimating provisional margins of exposure for data-poor chemicals using high-throughput computational methods
Source: Front Pharmacol. 2022 Oct 7;13:980747. doi: 10.3389/fphar.2022.980747 (PMC9586287; doi:10.3389/fphar.2022.980747)
Supplement: Supplementary file 1 [file DataSheet1.ZIP › Supplemental_Files_full_submitted/Exposure_Case_Study/Full_TTC_Analysis_MWL_Edits_Publication_REOrdered_v2.html]

Risk Prioritization Using Existing Tools and Methods


# Risk Prioritization Using Existing Tools and Methods

#### Chantel Nicolas and Matt Linakis

#### June 27, 2022

# OBJECTIVE

The purpose of this analysis is to demonstrate the utility of
exploiting existing computational approaches as part of a tiered
risk-based approach to prioritize ~45,000 of chemicals for further
analysis. One of the goals is to compare the results in this work to the
Becker et al work (EPA review) and the Paul Friedman et al work (2020).
Key differences between the two works is number of chemicals (45,000 vs
8,000 for Becker, 709 vs 448 for Paul Friedman) and methods (mixture of
free and fee-based tools vs use of free tools only). Thus, Leadscope was
not used in this work to identify carbamates based on structural
fingerprints; Toxtree v2.6.13 was used to determine carbamate structure.
Steroids were also not identified.

# METHODS

Here Toxtree was used in batch mode to process ~45,000 CERAPP
compounds across 5 different methods:  
1) Cramer rules – yields TTC Class I, II, or III  
2) Kroes TTC decision tree – yields ‘negligible risk’, ‘substance would
not be expected to be a safety concern’, or ‘risk assessment requires
compound-specific toxicity data’  
3) Structural Alerts for Functional Group Identification (ISSFUNC) –
yields codes associated with various functional groups

# DATA PROCESSING

Since the CERAPP dataset exceeded the max number of compounds per
batch, it was divided into several datasets and each was run across each
of the methods above in batch mode. A loop was run in order to integrate
1) results from all Toxtree methods for each batch and 2) results for
all batches together to arrive at a ~45,000 row dataset with respective
Toxtree observations. Here, the dataset will be further curated to
remove chemicals for which the Cramer decision tree (via Toxtree) does
not apply.

## Step 1: Load packages and functions.

Note, for some of these packages (e.g. fmcsR), you may need the
Bioconductor R Package Manager to download

## Step 2: Set input and output directories.

```
mainDir <- 
  # this.dir()
  #Use the commented codes instead if attempting to knit the document
  paste0(normalizePath(opts_knit$get()$root.dir))
subDir <- paste0(mainDir,"/Risk_Prioritization")
httkDir <- paste0(mainDir,"/Toxtree_HTTK")

source(paste0(mainDir,"/stat_smooth_func.R"))
```

## Step 3: Set up and import data

### Css Percentile Data based on httkpop

The first step is to read in the Css percentile data. We’ll go ahead
and do this for all 10 subpopulations.

```
#Set some basic parameters for which data set to use

#MWL Updated the MC analysis for the new version of httk
#Basically, instead of creating a population and reading it in
#to a calc_analytic_css function call,
#The calc_mc_css function now does all of that in 1 step, with
#a very long function call. Currently this takes a long time
#(on the order of hours) so it will just be read in from an
#.RDS file until this can be reformatted as a function as above
#for parallelization.
poormetab <- TRUE
fup.censored.dist <- TRUE
model <- '3compartmentss'
#List all the subpopulations
ExpoCast.groups <- c('Total',
                     'Age.6.11',
                     'Age.12.19',
                     'Age.20.65',
                     'Age.GT65',
                     'BMIgt30',
                     'BMIle30',
                     'Males',
                     'Females',
                     'ReproAgeFemale',
                     'Age.20.50.nonobese')
gendernum <- c(rep(list(NULL),7),
               list(list(Male=1000, Female=0)),
               list(list(Male=0, Female=1000)),
               list(list(Male=0, Female=1000)),
               list(NULL))
agelim<-c(list(c(0,79),
               c(6,11),
               c(12,19),
               c(20,65),
               c(66,79)),
          rep(list(c(0,79)),4),
          list(c(16,49)),
          list(c(20,50)))
bmi_category <- c(rep(list(c('Underweight',
                             'Normal',
                             'Overweight',
                             'Obese')),
                      5),
                  list('Obese', c('Underweight','Normal', 'Overweight')),
                  rep(list(c('Underweight',
                             'Normal',
                             'Overweight',
                             'Obese')),
                      3),
                  list(c('Underweight', 'Normal', 'Overweight')))
chem.dt <- as.data.table(httk::get_cheminfo(info=c('CAS', 'Compound')))
dat <- data.table(chemcas = character(), css95 = numeric(), ExpoCast.group = character())
j <- 1
# set.seed(1234)
# for(j in 1:length(ExpoCast.groups)){
#   for(i in 1:nrow(chem.dt)){
#     chemcas <- chem.dt$CAS[i]
#     css95 <- suppressWarnings(calc_mc_css(chem.cas = chem.dt$CAS[i],
#                                 output.units="uM",
#                                 invitro.mc.arg.list = list(adjusted.Funbound.plasma = TRUE,
#                                                            fup.censored.dist = fup.censored.dist, 
#                                                            poormetab = poormetab),
#                                 httkpop.generate.arg.list = list(method = "direct resampling",
#                                                                  gendernum = gendernum[[j]], 
#                                                                  agelim_years = agelim[[j]], 
#                                                                  weight_category = bmi_category[[j]]),
#                                 suppress.messages = T))[[1]]
#     ExpoCast.group = ExpoCast.groups[[j]]
#     dat <- rbind(dat,list(chemcas,css95,ExpoCast.group))
#   }
#   print(j) 
# }

dat <- readRDS(paste0(mainDir,"/new_httkpop_sim.rds"))
```

Get the compound names that correspond to each CAS.

```
setnames(chem.dt, 'CAS', 'chemcas')
dat <- merge(dat, chem.dt, by='chemcas')
httk_chems <- fread(paste0(httkDir,"/CCD-Batch-Search_2022-02-23_02_25_26.csv"), select = c(1:5))

length(unique(chem.dt$chemcas)) #998
```

```
## [1] 998
```

```
caslist <- chem.dt$chemcas
#Column names are Assay Endpoint, CASRN, Activity Call, Q, AC50, Emax, Log AC50, B, T, W, Data Type, Chemical Name.
```

### In Vitro ToxCast AC50 Values

Next, read in the ToxCast AC50 values.

```
# #Column names are Assay Endpoint, CASRN, Activity Call, Q, AC50, Emax, Log AC50,
# #B, T, W, Data Type, Chemical Name.
# #Replace names containing spaces with names without spaces.
# 
# #From Paul Friedman 2020 Analysis
# 
# # library(tcpl)
# # library(RMySQL)
# 
# # tcplConf(user='root', pass='z2zX141712', db='invitrodb', drvr='MySQL', host='localhost')
# # con <- dbConnect(drv = RMySQL::MySQL(), user="root",
# #                  password = "z2zX141712",
# #                  host = "localhost", database = invitrodb)
# 
# 
# # tc.dt <- as.data.table(tcplLoadChem(field = 'casn', val = caslist, exact = TRUE))
# # write.csv(tc.dt, "tc_dt.csv", row.names = F)
# # tc.dt.mc5 <- tcplPrepOtpt(tcplLoadData(lvl=5, type='mc', fld='spid', val=tc.dt$spid))
# # write.csv(tc.dt.mc5, "tc_dt_mc5.csv", row.names = F)
# 
# tc.dt <- as.data.table(read.csv(paste0(mainDir,"/tc_dt.csv")))
# tc.dt.mc5 <- as.data.table(read.csv(paste0(mainDir,"/tc_dt_mc5.csv")))
# tc.dt.sub <- tc.dt.mc5[which(tc.dt.mc5$hitc == 1)]
# 
# total.casn <-unique(tc.dt.mc5$casn) #933
# toxcast.casn <- unique(tc.dt.sub$casn) #915
# negatives.casn <- setdiff(total.casn, toxcast.casn) #18
# 
# #Min AC50 approximation from ToxCast
# toxcast.m4id.list <- unique(tc.dt.sub[,m4id])
# 
# # mc7 <- dbGetQuery(con, "SELECT 
# #                   mc7.*
# #                   FROM
# #                   invitrodb.mc7;") %>% 
# #   data.table()
# # write.csv(mc7,"mc7.csv", row.names = F)
# 
# mc7 <- as.data.table(read.csv(paste0(mainDir,"/mc7.csv")))
# mc7 <- mc7[m4id %in% toxcast.m4id.list]
# positive.spids <- tc.dt.sub[,spid]
# 
# # mc6 <- tcplPrepOtpt(tcplLoadData(lvl=6, fld='spid', val=positive.spids, type='mc'))
# # write.csv(mc6, "mc6.csv", row.names = F)
# 
# mc6 <- as.data.table(read.csv(paste0(mainDir,"/mc6.csv")))
# 
# setDT(mc6)
# mc6_mthds <- mc6[ , .( mc6_mthd_id = paste(mc6_mthd_id, collapse=",")), by = m4id]
# mc6_flags <- mc6[ , .( flag = paste(flag, collapse=";")), by = m4id]
# 
# tc.dt.sub$mc6_flags <- mc6_mthds$mc6_mthd_id[match(tc.dt.sub$m4id, mc6_mthds$m4id)]
# tc.dt.sub[, flag.length := ifelse(!is.na(mc6_flags), count.fields(textConnection(mc6_flags), sep =','), NA)]
# tc.dt.sub$hitpct <- mc7$hit_pct[match(tc.dt.sub$m4id, mc7$m4id)]
# tc.dt.sub$modl_ga_delta <- mc7$modl_ga_delta[match(tc.dt.sub$m4id, mc7$m4id)]
# 
# #Filtering ToxCast
# 
# tc.dt.sub[flag.length < 3, use.me := 1]
# tc.dt.sub[is.na(flag.length), use.me := 1]
# tc.dt.sub[hitpct <= 0.500 & flag.length >= 3, use.me := 0]
# tc.dt.sub[fitc %in% c(36,45), use.me := 0]
# tc.dt.sub[,length.use.curves:= sum(use.me, na.rm=TRUE), by=list(casn)]
# length(unique(tc.dt.sub[length.use.curves==0]$chid))
# nocurves <- tc.dt.sub$casn[tc.dt.sub$length.use.curves==0] 
# 
# filter.tc.dt.sub <- tc.dt.sub[use.me==1] #down to 106202 curves from 119886 hitc1 curves (46735/119886)*100
# length(unique(filter.tc.dt.sub$casn)) #910 casrn
# filter.tc.dt.sub[, hitcsum := sum(hitc), by=list(casn)]
# filter.tc.dt.sub[, modl_ga_uM := ifelse(!is.na(modl_ga), 10^modl_ga, NA)]
# filter.tc.dt.sub[, min_modl_ga_uM := min(modl_ga_uM, na.rm=TRUE), by=list(chid)] 
# 
# # negatives.chemdb<-tcplLoadChem(field='casn', val=nocurves, exact=TRUE)
# # write.csv(negatives.chemdb,"negatives_chemdb.csv", row.names = F)
# 
# negatives.chemdb <- as.data.table(read.csv(paste0(mainDir,"/negatives_chemdb.csv")))
# negatives.spids<-negatives.chemdb$spid
# 
# # negatives.mc5<-tcplPrepOtpt(tcplSubsetChid(tcplLoadData(lvl=5, fld='spid', val=negatives.spids, type='mc')))
# # write.csv(negatives.mc5,"negatives_mc5.csv", row.names = F)
# 
# negatives.mc5 <- as.data.table(read.csv(paste0(mainDir,"/negatives_mc5.csv")))
# negatives.mc5 <- unique(negatives.mc5[,list(chid,casn,chnm,code,hitc)])
# negatives.mc5 <- negatives.mc5[!hitc==1]
# negatives.casn <- negatives.mc5[,casn]
# 
# toxcast.master <- merge(filter.tc.dt.sub,
#                                 negatives.mc5,
#                                 by=c('chid','casn','chnm','code'),
#                                 all.x=TRUE,
#                                 all.y=TRUE)
# # Keep only the rows of ToxCast data where the chemical was judged "Active" in an assay. 
# toxcast.master[casn %in% negatives.casn, length.use.curves := 0]
# toxcast.master[casn %in% negatives.casn, hitcsum := 0]
# toxcast.master[casn %in% negatives.casn, modl_ga := 2]
# toxcast.master[casn %in% negatives.casn, modl_ga_uM := 100]
# toxcast.master[casn %in% negatives.casn, min_modl_ga_uM := 100]
# toxcast.master[,c('hitc.y'):= NULL]
# 
# length(unique(toxcast.master$casn)) #915
# # We summarize the distribution of AC50 values across all assays for each chemical by taking several percentiles.
# ac50pct <- toxcast.master[, as.list(quantile(modl_ga_uM, probs=c(0,0.05,0.1,0.25,0.5,0.75,0.9,0.95,1), 
#                                                na.rm=TRUE)),keyby=casn]
# ac50pct$hitcsum<-toxcast.master$hitcsum[match(ac50pct$casn, toxcast.master$casn)]
# ac50pct$min_modl_ga_uM <- toxcast.master$min_modl_ga_uM[match(ac50pct$casn, toxcast.master$casn)]
# ac50pct$chnm <- toxcast.master$chnm[match(ac50pct$casn, toxcast.master$casn)]
# pctnames <- grep(names(ac50pct), pattern='%',value=TRUE)
# # By default, the columns of `ac50pct` have names like "5%", "10%", etc. The "%" sign interferes with `data.table`'s 
# # syntax for referring to columns. So we replace names like "5%" with names like "AC50p5", to indicate that the column 
# # contains AC50 values, percentile 5. To do this, use regular expressions.
# setnames(ac50pct,
#          pctnames,
#          gsub(x=pctnames, 
#               pattern='(\\d{1,3})\\%', 
#               replacement='AC50p\\1', 
#               perl=TRUE) 
#          )
# ac50pct[,avg.dist := AC50p5 - min_modl_ga_uM]
# setnames(ac50pct, 'casn', 'chemcas')
# # write.csv(ac50pct,"ac50pct.csv", row.names = F)
```

### Compute OEDs from AC50 and Css95

Now we are ready to compute OEDs. Let’s compute one OED corresponding
to each ToxCast AC50, using the 95th percentile Css for all of them.

```
#merge in the AC50 percentile data
ac50pct <- as.data.table(read.csv(paste0(mainDir,"/ac50pct.csv")))
m.tmp <- merge(dat,ac50pct,by='chemcas')
#find column names beginning with "AC50"
#to use for naming the OED columns
ac50names <- grep(x=names(m.tmp),
                  pattern='^AC50', 
                  value=TRUE,
                  perl=TRUE)
#Compute OEDs
m.tmp[, (paste('oed', ac50names, sep='.')):=lapply(.SD,
                                                   function(x) x/m.tmp[, css95]),
      .SDcols=ac50names] #unitless * 1 mg/kg/day (Ring et al, 2017, https://www.ncbi.nlm.nih.gov/pmc/articles/PMC6116525/) = mg/kg/day
```

Then, take care of some housekeeping. Replace a couple of very long
chemical names with shorter names, for better display.

```
#Shorten a couple of compound names for display
m.tmp[Compound=="O-ethyl o-(p-nitrophenyl) phenylphosphonothioate",
      Compound:="Phosphonothioic acid"]
m.tmp[Compound=="4-(1,1,3,3-tetramethylbutyl)phenol",
      Compound:="p-tert-Octylphenol"]
length(unique(m.tmp$chemcas)) #with invitrodbv3.4: 915 out of 998!
```

```
## [1] 915
```

The remaining 915 chemicals were processed on the NCCT Chemistry
Dashboard to get their QSAR-ready structures. The ones that have
QSAR-ready structures were processed in the Toxtree app to obtain
TTCs

### Import SEEM3 Exposure estimates

Here SEEM3 exposure estimates from Ring 2019 supplemental material
are imported. Some additional code is also left over in order to fill
missing estimates with either SEEM2 estimates or averaged SEEM estimates
from the online dashboard.

```
# seem3db <- as.data.table(read.csv(paste0(mainDir,"/SupTable-all.chem.preds-2018-11-28.csv"))) #From Ring 2019 et al. supplemental material
# seem3db.sub <- seem3db[match(chem.dt$chemcas,seem3db$CAS)]
# seem3db.sub <- seem3db.sub[!is.na(CAS)] #23 not in the database
# write.csv(seem3db.sub,paste0(mainDir,"/seem3db_sub.csv"),row.names = F)

seem3db.sub <- as.data.table(read.csv(paste0(mainDir,"/seem3db_sub.csv")))
seem3db.pos <- seem3db.sub[!is.na(seem3db.sub$seem3.u95)] #750 with results
seem3db.miss <- seem3db.sub[is.na(seem3db.sub$seem3.u95)] #225 with no upper 95% level

#Replace CERAPP SEEM2 Exposure Estimates with SEEM3 estimates where possible
CERAPP_HeuristicExposure <- as.data.table(read.csv(paste0(mainDir,"/CERAPP_HeuristicExposure.csv")))
CERAPP_HeuristicExposure <- CERAPP_HeuristicExposure[!duplicated(Substance_CASRN)]
# seem3db.CERAPP <- seem3db[match(CERAPP_HeuristicExposure$Substance_CASRN,seem3db$CAS)]
# write.csv(seem3db.CERAPP,paste0(mainDir,"/seem3db_cerapp.csv"),row.names = F)

seem3db.CERAPP <- as.data.table(read.csv(paste0(mainDir,"/seem3db_cerapp.csv")))
CERAPP_HeuristicExposure$Total.Upper95[!is.na(seem3db.CERAPP$seem3.u95)] <- seem3db.CERAPP$seem3.u95[match(CERAPP_HeuristicExposure$Substance_CASRN,seem3db.CERAPP$CAS) & !is.na(seem3db.CERAPP$seem3.u95)]

#If you are looking to crawl through the EPA tables for data that IS available for SEEM3 (all but total),
#then you can run the below script. Note that the chromever may need to change based on what versions you
#have, run binman::list_versions("chromedriver") and choose the version that is less than or equal to your
#current Google Chrome version.

# library(rvest)
# library(dplyr)
# library(RSelenium)
# driver = rsDriver(browser = c("chrome"), chromever = '100.0.4896.60')
# remDr <- driver[["client"]]
# testouter <- list()
# testinner <- list()
# 
# for(i in 1:nrow(seem3db.miss)){
#   dtxsid <- seem3db.miss$dsstox_substance_id[i]
#   url <- paste0('https://comptox.epa.gov/dashboard/chemical/exposure-predictions/',dtxsid)
#   remDr$navigate(url)
#   for(j in 1:9){
#     testinner[[j]] = remDr$getPageSource()[[1]] %>% 
#     rvest::read_html() %>% rvest::html_nodes(xpath = paste0( '/html/body/div/div/div/div[5]/div[2]/main/div/div[3]/div/div/div[2]/div/div[2]/div[2]/div[3]/div[2]/div/div/div[',j,']/div[3]/div/span/span')) %>% 
#     rvest::html_text()
#   }
#   testouter[[i]] <- testinner
# }
# testmat <- as.data.table(do.call(rbind, testouter))
# names(testmat) <- c("Age_6_11","Age_12_19","Age_20_65","Age_65_up","BMI_lt_30","BMI_gt_30","Females","Males","Repro_Age_Females")
# testmat <- as.data.table(sapply( testmat, as.numeric ))
# testmat$Avg <- rowMeans(testmat,na.rm=T)
# testmat$Chem <- seem3db.miss$Substance_Name
# testmat$CAS <- seem3db.miss$CAS
# testmat$DTXSID <- seem3db.miss$dsstox_substance_id
# seem3avg <- testmat
# write.csv(seem3avg,"Missing_SEEM3_U95.csv", row.names = F)

seem3avg <- as.data.table(read.csv(paste0(mainDir,"/Missing_SEEM3_U95.csv")))

#For the ones that are missing U95 values, default first to filling in from old SEEM2 estimates
# seem3db.miss$seem3.u95 <- CERAPP_HeuristicExposure$Total.Upper95[match(seem3db.miss$CAS,CERAPP_HeuristicExposure$Substance_CASRN)] #Adds an additional 205 chemicals
# #Then fill in the rest by taking averages of other populations pulled from CompTox Chemicals Dashboard
# seem3db.miss$seem3.u95[is.na(seem3db.miss$seem3.u95)] <- seem3avg$Avg[is.na(seem3db.miss$seem3.u95)] #Adds another 14 chemicals, leaving 6 total with no SEEM data available

# #Merge SEEM3 with SMILES for Kroes Estimation
kroesinput <- merge(seem3db.sub,httk_chems, by.x = "dsstox_substance_id",by.y = "DTXSID")
kroesinput$NAME <- kroesinput$Substance_Name
kroesinput$SMILES <- kroesinput$QSAR_READY_SMILES
kroesinput$DailyIntake <- kroesinput$seem3*60*1000
kroesinput <- select(kroesinput,NAME,CAS,SMILES,DailyIntake)
print(nrow(kroesinput))
```

```
## [1] 975
```

```
# write.csv(kroesinput,paste0(httkDir,"/kroesinput.csv"),row.names = F, quote = F)

#Finally, combine back into a full dataset
# seem3db.httk <- rbind(seem3db.pos,seem3db.miss)
seem3db.httk <- seem3db.pos
```

### Import In Vivo date (NOAELs) from ToxValDB

Data are then imported from ToxValDB (https://comptox.epa.gov/dashboard/about for most updated
version). TRI-CHIP can also be used to pull in NOAELs from IRIS if
desired.

```
#For the smaller, TRI-CHIP database
# # TRICHIP_IRIS_NOAEL <- fread(paste0(mainDir,"/TRI_CHIP_IRIS_NOAEL_2022.csv"), select = c(2:3,8,12,14))
# # setnames(TRICHIP_IRIS_NOAEL,c("casrn","Name","NOAEL","Species","Categories"))
# # TRICHIP_IRIS_NOAEL$NOAEL <- as.numeric(TRICHIP_IRIS_NOAEL$NOAEL)
# 
# #For the larger ToxValDB (https://comptox.epa.gov/dashboard/about), filtering similar to Paul-Friedman 2020, though only with NOAELs
# toxvaldb <- as.data.table(read.csv(paste0(mainDir,"/toxval_all_with_references_dev_toxval_v9_1_2021-06-01.csv")))
# # toxvaldb <- toxvaldb[toxval_type %in% c("LOAEL","LOEL","NOAEL","NOEL")] #
# toxvaldb <- toxvaldb[toxval_type %in% c("NOAEL")]
# # toxvaldb <- toxvaldb[toxval_units %in% c("mg/day","mg/kg","mg/kg-day")]
# toxvaldb <- toxvaldb[toxval_units %in% c("mg/kg-day")]
# toxvaldb <- toxvaldb[exposure_route == "oral"]
# #choose the 5th percentile using type 2 algorithm per Paul Friedman 2020
# toxvaldb.indivchem <- aggregate(toxval_numeric~casrn, data = toxvaldb, FUN = 'quantile',probs = c(0.05), type = 2)
# # write.csv(toxvaldb.indivchem,paste0(mainDir,"/toxval_indiv.csv"),row.names = F)

toxvaldb.indivchem <- as.data.table(read.csv(paste0(mainDir,"/toxval_indiv.csv")))
# length(intersect(seem3db.httk$CAS, toxvaldb$casrn))
```

## Step 4a: Process HTTK Toxtree (TTC) data.

Toxtree data was re-run with additional chems on 2-23-2022 MWL

```
# merged.data <- NULL
# mergehttkSDF <- function(){
# sdfset.Cramer  <- read.SDFset(paste0(httkDir,"/Toxtree_HTTK_Cramer_2022_v2.sdf"))
# sdfset.Cramer.Ext  <- read.SDFset(paste0(httkDir,"/Toxtree_HTTK_Cramer_Ext_2022_v2.sdf"))
# sdfset.Kroes  <- read.SDFset(paste0(httkDir,"/Toxtree_HTTK_Kroes_2022_v2.sdf"))
# sdfset.Carcinogenicity  <- read.SDFset(paste0(httkDir,"/Toxtree_HTTK_Carcinogenicity_ISS_2022_v2.sdf"))
# sdfset.Ames  <- read.SDFset(paste0(httkDir,"/Toxtree_HTTK_Ames_ISS_2022_v2.sdf"))
# sdfset.Functional  <- read.SDFset(paste0(httkDir,"/Toxtree_HTTK_Func_ISS_2022_v2.sdf"))
# valid.Cramer.Ext <- validSDF(sdfset.Cramer.Ext) # Identifies invalid SDFs in SDFset objects
# valid.Cramer <- validSDF(sdfset.Cramer) # Identifies invalid SDFs in SDFset objects
# valid.Kroes <- validSDF(sdfset.Kroes) # Identifies invalid SDFs in SDFset objects
# valid.Carcinogenicity<- validSDF(sdfset.Carcinogenicity) # Identifies invalid SDFs in SDFset objects
# valid.Ames <- validSDF(sdfset.Ames) # Identifies invalid SDFs in SDFset objects
# valid.Functional <- validSDF(sdfset.Functional) # Identifies invalid SDFs in SDFset objects
# sdfset.Cramer <- sdfset.Cramer[valid.Cramer] # Removes invalid SDFs, if there are any
# sdfset.Cramer.Ext <- sdfset.Cramer.Ext[valid.Cramer.Ext] # Removes invalid SDFs, if there are any
# sdfset.Kroes <- sdfset.Kroes[valid.Kroes] # Removes invalid SDFs, if there are any
# sdfset.Carcinogenicity <- sdfset.Carcinogenicity[valid.Carcinogenicity] # Removes invalid SDFs, if there are any
# sdfset.Ames <- sdfset.Ames[valid.Ames] # Removes invalid SDFs, if there are any
# sdfset.Functional <- sdfset.Functional[valid.Functional] # Removes invalid SDFs, if there are any
# blockmatrix.Cramer <- datablock2ma(datablocklist=datablock(sdfset.Cramer)) # Converts data block to matrix
# blockmatrix.Cramer <- blockmatrix.Cramer[,-1] #Removes the CDK:Title column as no title was assigned, MWL 2-23-22
# blockmatrix.Cramer <- blockmatrix.Cramer[!duplicated(blockmatrix.Cramer[,2]),]
# print(dim(blockmatrix.Cramer))
# blockmatrix.Cramer.Ext <- datablock2ma(datablocklist=datablock(sdfset.Cramer.Ext)) # Converts data block to matrix
# blockmatrix.Cramer.Ext <- blockmatrix.Cramer.Ext[,-1] #Removes the CDK:Title column as no title was assigned, MWL 2-23-22
# blockmatrix.Cramer.Ext <- blockmatrix.Cramer.Ext[!duplicated(blockmatrix.Cramer.Ext[,2]),]
# print(dim(blockmatrix.Cramer.Ext))
# blockmatrix.Kroes <- datablock2ma(datablocklist=datablock(sdfset.Kroes)) # Converts data block to matrix
# blockmatrix.Kroes <- blockmatrix.Kroes[,-1] #Removes the CDK:Title column as no title was assigned, MWL 2-23-22
# blockmatrix.Kroes <- blockmatrix.Kroes[!duplicated(blockmatrix.Kroes[,2]),]
# print(dim(blockmatrix.Kroes))
# blockmatrix.Carcinogenicity <- datablock2ma(datablocklist=datablock(sdfset.Carcinogenicity)) # Converts data block to matrix
# blockmatrix.Carcinogenicity <- blockmatrix.Carcinogenicity[,-1] #Removes the CDK:Title column as no title was assigned, MWL 2-23-22
# blockmatrix.Carcinogenicity <- blockmatrix.Carcinogenicity[!duplicated(blockmatrix.Carcinogenicity[,2]),]
# print(dim(blockmatrix.Carcinogenicity))
# blockmatrix.Ames <- datablock2ma(datablocklist=datablock(sdfset.Ames)) # Converts data block to matrix
# blockmatrix.Ames <- blockmatrix.Ames[,-1] #Removes the CDK:Title column as no title was assigned, MWL 2-23-22
# blockmatrix.Ames <- blockmatrix.Ames[!duplicated(blockmatrix.Ames[,2]),]
# print(dim(blockmatrix.Ames))
# blockmatrix.Functional <- datablock2ma(datablocklist=datablock(sdfset.Functional)) # Converts data block to matrix
# blockmatrix.Functional <- blockmatrix.Functional[,-1] #Removes the CDK:Title column as no title was assigned, MWL 2-23-22
# blockmatrix.Functional <- blockmatrix.Functional[!duplicated(blockmatrix.Functional[,2]),]
# print(dim(blockmatrix.Functional))
# colnames(blockmatrix.Carcinogenicity)[8] <-"For a better assessment a QSAR calculation could be applied_Carc"
# colnames(blockmatrix.Carcinogenicity)[11] <- "Error when applying the decision tree_Carc"
# colnames(blockmatrix.Ames)[6] <- "For a better assessment a QSAR calculation could be applied_Ames"
# colnames(blockmatrix.Ames)[7] <- "Error when applying the decision tree_Ames"
# HTTK_Toxtree <- merge(blockmatrix.Cramer,blockmatrix.Cramer.Ext, by = c("NAME","CAS","SMILES","DailyIntake"))
# print(dim(HTTK_Toxtree))
# HTTK_Toxtree <- merge(HTTK_Toxtree,blockmatrix.Kroes, by = c("NAME","CAS","SMILES","DailyIntake"))
# print(dim(HTTK_Toxtree))
# HTTK_Toxtree <- merge(HTTK_Toxtree,blockmatrix.Carcinogenicity[,c(2,5)], by = c("CAS"))
# print(dim(HTTK_Toxtree))
# HTTK_Toxtree <- merge(HTTK_Toxtree,blockmatrix.Ames[,c(2,5)], by = c("CAS"))
# print(dim(HTTK_Toxtree))
# HTTK_Toxtree <- merge(HTTK_Toxtree,blockmatrix.Functional[,c(2,94,36)], by = c("CAS"))
# merged.data
# merged.data <- rbind(merged.data,HTTK_Toxtree)
# }
# HTTK_Toxtree <- mergehttkSDF()
# write.csv(HTTK_Toxtree,paste0(mainDir,"/HTTK_Toxtree_2.csv"), row.names = F)

HTTK_Toxtree <- as.data.table(read.csv(paste0(mainDir,"/HTTK_Toxtree_3.csv"), check.names = F))
```

## Step 4b: Process CERAPP Toxtree (TTC) data.

This uses the Toxtree results generated from the original analysis
(31 batches in total) since an updated CERAPP list was not found.
Because this takes a while to run, it is just loaded from a .csv file,
but if an updated list (or a new list of chemicals) is desired, the code
that’s been commented out can simply be uncommented and the dataset can
be generated from the updated/new .sdf files.

```
# ptm <- proc.time()
# merged.data <- NULL
# mergeSDF <- function(){
#     for (i in 0:30) {
#     print(i)
#     sdfset.Cramer  <- read.SDFset(paste0(cerappDir,"/Batch_",i,"_Cerapp_TTC_Cramer.sdf"))
#     sdfset.Cramer.Ext  <- read.SDFset(paste0(cerappDir,"/Batch_",i,"_Cerapp_TTC_Cramer_Ext.sdf"))
#     sdfset.Kroes  <- read.SDFset(paste0(cerappDir,"/Batch_",i,"_Cerapp_Kroes.sdf"))
#     sdfset.Carcinogenicity  <-     read.SDFset(paste0(cerappDir,"/Batch_",i,"_Cerapp_Carcinogenicity_ISS.sdf"))
#     sdfset.Ames  <- read.SDFset(paste0(cerappDir,"/Batch_",i,"_Cerapp_Ames_ISS.sdf"))
#     sdfset.Functional  <- read.SDFset(paste0(cerappDir,"/Batch_",i,"_Cerapp_Func_ISS.sdf"))
#     valid.Cramer.Ext <- validSDF(sdfset.Cramer.Ext) # Identifies invalid SDFs in SDFset objects
#     valid.Cramer <- validSDF(sdfset.Cramer) # Identifies invalid SDFs in SDFset objects
#     valid.Kroes <- validSDF(sdfset.Kroes) # Identifies invalid SDFs in SDFset objects
#     valid.Carcinogenicity<- validSDF(sdfset.Carcinogenicity) # Identifies invalid SDFs in SDFset objects
#     valid.Ames <- validSDF(sdfset.Ames) # Identifies invalid SDFs in SDFset objects
#     valid.Functional <- validSDF(sdfset.Functional) # Identifies invalid SDFs in SDFset objects
#     sdfset.Cramer <- sdfset.Cramer[valid.Cramer] # Removes invalid SDFs, if there are any
#     sdfset.Cramer.Ext <- sdfset.Cramer.Ext[valid.Cramer.Ext] # Removes invalid SDFs, if there are any
#     sdfset.Kroes <- sdfset.Kroes[valid.Kroes] # Removes invalid SDFs, if there are any
#     sdfset.Carcinogenicity <- sdfset.Carcinogenicity[valid.Carcinogenicity] # Removes invalid SDFs, if there are any
#     sdfset.Ames <- sdfset.Ames[valid.Ames] # Removes invalid SDFs, if there are any
#     sdfset.Functional <- sdfset.Functional[valid.Functional] # Removes invalid SDFs, if there are any
#     blockmatrix.Cramer <- datablock2ma(datablocklist=datablock(sdfset.Cramer)) # Converts data block to matrix
#     blockmatrix.Cramer <- blockmatrix.Cramer[!duplicated(blockmatrix.Cramer[,1]),]
#     # print(dim(blockmatrix.Cramer))
#     blockmatrix.Cramer.Ext <- datablock2ma(datablocklist=datablock(sdfset.Cramer.Ext)) # Converts data block to matrix
#     blockmatrix.Cramer.Ext <- blockmatrix.Cramer.Ext[!duplicated(blockmatrix.Cramer.Ext[,1]),]
#     # print(dim(blockmatrix.Cramer.Ext))
#     blockmatrix.Kroes <- datablock2ma(datablocklist=datablock(sdfset.Kroes)) # Converts data block to matrix
#     blockmatrix.Kroes <- blockmatrix.Kroes[!duplicated(blockmatrix.Kroes[,1]),]
#     # print(dim(blockmatrix.Kroes))
#     blockmatrix.Carcinogenicity <- datablock2ma(datablocklist=datablock(sdfset.Carcinogenicity)) # Converts data block to matrix
#     blockmatrix.Carcinogenicity <- blockmatrix.Carcinogenicity[!duplicated(blockmatrix.Carcinogenicity[,1]),]
#     # print(dim(blockmatrix.Carcinogenicity))
#     blockmatrix.Ames <- datablock2ma(datablocklist=datablock(sdfset.Ames)) # Converts data block to matrix
#     blockmatrix.Ames <- blockmatrix.Ames[!duplicated(blockmatrix.Ames[,1]),]
#     # print(dim(blockmatrix.Ames))
#     blockmatrix.Functional <- datablock2ma(datablocklist=datablock(sdfset.Functional)) # Converts data block to matrix
#     blockmatrix.Functional <- blockmatrix.Functional[!duplicated(blockmatrix.Functional[,1]),]
#     # print(dim(blockmatrix.Functional))
#     colnames(blockmatrix.Carcinogenicity)[12] <-"For a better assessment a QSAR calculation could be applied_Carc"
#     colnames(blockmatrix.Carcinogenicity)[15] <- "Error when applying the decision tree_Carc"
#     colnames(blockmatrix.Ames)[10] <- "For a better assessment a QSAR calculation could be applied_Ames"
#     colnames(blockmatrix.Ames)[11] <- "Error when applying the decision tree_Ames"
#     block.merge0 <- merge(blockmatrix.Cramer,blockmatrix.Cramer.Ext, by = c("casrn","SMILES","dsstox_substance_id","MW","Batch"))
#     # print(dim(block.merge0))
#     block.merge1 <- merge(block.merge0,blockmatrix.Kroes, by = c("casrn","SMILES","dsstox_substance_id","MW","Batch"))
#     # print(dim(block.merge1))
#     block.merge2 <- merge(block.merge1,blockmatrix.Carcinogenicity[,c(1:16)], by = c("casrn","SMILES","dsstox_substance_id","MW","Batch"))
#     # print(dim(block.merge2))
#     block.merge3 <- merge(block.merge2,blockmatrix.Ames[,c(1:7,10:12)], by = c("casrn","SMILES","dsstox_substance_id","MW","Batch"))
#     # print(dim(block.merge3))
#     # browser()
#     block.merge4 <- merge(block.merge3,blockmatrix.Functional[,c(1:7,38,192,37,95,198)], by = c("casrn","SMILES","dsstox_substance_id","MW","Batch"))
#     # print
#     (dim(block.merge4))
#     #Kept or excluded various columns from .Carcinogenicity and .Ames
#     #The following were duplicated in both and were assumed to be unique so "_Carc" or "_Ames" was added: c("For a better assessment a QSAR calculation could be applied." , "Error when applying the decision tree.")
#     #The following were duplicated in both and were assumed to be duplicates and so one was kept: c("ar-N=CH2", "aN=Na", "QSAR13 applicable?")
#     merged.data
#     merged.data <- rbind(merged.data,block.merge4)
#     assign(paste0("Batch_", i), merged.data)
#   }
#     merged.data
# }
# CERAPP_Toxtree_Results <- mergeSDF()
# proc.time() - ptm
# write.csv(CERAPP_Toxtree_Results,paste0(mainDir,"/CERAPP_Toxtree_Results.csv"), row.names = F)

CERAPP_Toxtree_Results <- as.data.table(read.csv(paste0(mainDir,"/CERAPP_Toxtree_Results.csv"), check.names = F))
```

## Step 5a: Assign Numeric TTC values to HTTK chemicals based on Toxtree results.

Track number of compounds that are: 1) Kroes requires chemical
specific information for determining TTC class  
2) Anti-cholinesterase inhibiting (Anti-ChE Organophosphate ester or
Carbamate Ester) 3) Cramer Class III 4) Cramer Class II 5) Cramer Class
I

```
httk_oed_SMILES_NHANES <- fread(paste0(httkDir,"/CCD-Batch-Search_2022-02-23_02_25_26.csv"), select = c(1:5))
HTTK.Toxtree <- merge(httk_oed_SMILES_NHANES,HTTK_Toxtree, by.x = c("QSAR_READY_SMILES"),by.y = c("SMILES"))
print(dim(HTTK.Toxtree)) #985
```

```
## [1] 1028   18
```

```
HTTK.Toxtree$Genotoxic <- ifelse(HTTK.Toxtree$`Structural Alert for genotoxic carcinogenicity` == "YES" | HTTK.Toxtree$`Structural Alert for S. typhimurium  mutagenicity` == "YES", "YES", "NO")
HTTK.Toxtree$Anti_ChE <- ifelse(HTTK.Toxtree$FG52_2 == "YES" | HTTK.Toxtree$FG81_2 == "YES", "YES", "NO")

# seem3db <- as.data.table(read.csv("SupTable-all.chem.preds-2018-11-28.csv"))

#Get m.tmp to look like Cerapp_heuristic total upper and median 

HTTK.Toxtree$Exposure.Total.Upper95 <- seem3db.httk$seem3.u95[match(HTTK.Toxtree$DTXSID,seem3db.httk$dsstox_substance_id)]
HTTK.Toxtree$Exposure.Total.Median <- seem3db.httk$seem3[match(HTTK.Toxtree$DTXSID,seem3db.httk$dsstox_substance_id)]

HTTK.Toxtree$oed.AC50p5 <-  m.tmp$oed.AC50p5[match(HTTK.Toxtree$INPUT,m.tmp$chemcas)]
HTTK.Toxtree$oed.AC50p50 <-  m.tmp$oed.AC50p50[match(HTTK.Toxtree$INPUT,m.tmp$chemcas)]
HTTK.Toxtree$oed.AC50p95 <-  m.tmp$oed.AC50p95[match(HTTK.Toxtree$INPUT,m.tmp$chemcas)]

# HTTK.Toxtree$NOAEL <- as.numeric(TRICHIP_IRIS_NOAEL$NOAEL[match(HTTK.Toxtree$INPUT,TRICHIP_IRIS_NOAEL$casrn)]) 
HTTK.Toxtree$NOAEL <- as.numeric(toxvaldb.indivchem$toxval_numeric[match(HTTK.Toxtree$INPUT,toxvaldb.indivchem$casrn)]) 

HTTK.Toxtree <- HTTK.Toxtree[-which(is.na(HTTK.Toxtree$Exposure.Total.Upper95) == TRUE),]
HTTK.Toxtree <- HTTK.Toxtree[!duplicated(HTTK.Toxtree$DTXSID),]

#For the main analysis, we're going to ignore the genotoxic flag. It's exceptionally
#conservative and skews the results quite a bit. Note, this does drop the n from
#45032 to 45012
#Separate out genotox analysis as gHTTK.Toxtree
gHTTK.Toxtree <- HTTK.Toxtree

#Original Analysis
HTTK.Toxtree$TTC_ug.kg.d <-   ifelse(HTTK.Toxtree$Anti_ChE == "YES", 0.3, ifelse(HTTK.Toxtree$`Cramer rules, with extensions` == "High (Class III)", 1.5, ifelse(HTTK.Toxtree$`Cramer rules, with extensions` == "Intermediate (Class II)", 9, ifelse(HTTK.Toxtree$`Cramer rules, with extensions` == "Low (Class I)", 30,NA))))

HTTK.Toxtree$TTC_Class <- ifelse(HTTK.Toxtree$TTC_ug.kg.d == 0.0025, "Genotoxic", ifelse(HTTK.Toxtree$TTC_ug.kg.d == 0.3, "Anti-cholinesterase", ifelse(HTTK.Toxtree$TTC_ug.kg.d == 1.5, "Cramer Class III", ifelse(HTTK.Toxtree$TTC_ug.kg.d == 9, "Cramer Class II", ifelse(HTTK.Toxtree$TTC_ug.kg.d == 30, "Cramer Class I",NA)))))

HTTK.Toxtree$TTC_MoE <- (0.001*HTTK.Toxtree$TTC_ug.kg.d)/HTTK.Toxtree$Exposure.Total.Upper95 #divide by 1000 to get mg/kg/d, already safety factor 100 here
HTTK.Toxtree$OED_MoE <- (HTTK.Toxtree$oed.AC50p5)/HTTK.Toxtree$Exposure.Total.Upper95
# HTTK.Toxtree$NOAEL_MoE <- (0.01*HTTK.Toxtree$NOAEL)/HTTK.Toxtree$Exposure.Total.Upper95 #divide by safety factor of 100
HTTK.Toxtree$NOAEL_MoE <- (HTTK.Toxtree$NOAEL)/HTTK.Toxtree$Exposure.Total.Upper95

HTTK.Toxtree <- HTTK.Toxtree[-which(is.na(HTTK.Toxtree$OED_MoE)==TRUE),]

#Genotox analysis
gHTTK.Toxtree$TTC_ug.kg.d <- ifelse(gHTTK.Toxtree$Genotoxic == "YES", 0.0025, ifelse(gHTTK.Toxtree$Anti_ChE == "YES", 0.3, ifelse(gHTTK.Toxtree$`Cramer rules, with extensions` == "High (Class III)", 1.5, ifelse(gHTTK.Toxtree$`Cramer rules, with extensions` == "Intermediate (Class II)", 9, ifelse(gHTTK.Toxtree$`Cramer rules, with extensions` == "Low (Class I)", 30,NA)))))

gHTTK.Toxtree$TTC_Class <- ifelse(gHTTK.Toxtree$TTC_ug.kg.d == 0.0025, "Genotoxic", ifelse(gHTTK.Toxtree$TTC_ug.kg.d == 0.3, "Anti-cholinesterase", ifelse(gHTTK.Toxtree$TTC_ug.kg.d == 1.5, "Cramer Class III", ifelse(gHTTK.Toxtree$TTC_ug.kg.d == 9, "Cramer Class II", ifelse(gHTTK.Toxtree$TTC_ug.kg.d == 30, "Cramer Class I",NA)))))


gHTTK.Toxtree$TTC_MoE <- (0.001*gHTTK.Toxtree$TTC_ug.kg.d)/gHTTK.Toxtree$Exposure.Total.Upper95 #divide by 1000 to get mg/kg/d, already safety factor 100 here
gHTTK.Toxtree$OED_MoE <- (gHTTK.Toxtree$oed.AC50p5)/gHTTK.Toxtree$Exposure.Total.Upper95
# gHTTK.Toxtree$NOAEL_MoE <- (0.01*gHTTK.Toxtree$NOAEL)/gHTTK.Toxtree$Exposure.Total.Upper95 #divide by safety factor of 100
gHTTK.Toxtree$NOAEL_MoE <- (gHTTK.Toxtree$NOAEL)/gHTTK.Toxtree$Exposure.Total.Upper95

gHTTK.Toxtree <- gHTTK.Toxtree[-which(is.na(gHTTK.Toxtree$OED_MoE)==TRUE),]


dim(HTTK.Toxtree) #709
```

```
## [1] 709  31
```

```
dim(gHTTK.Toxtree) #709
```

```
## [1] 709  31
```

## Step 5b: Assign Numeric TTC values based to CERAPP chemicals on Toxtree results.

Track number of compounds that are: 1) Kroes requires chemical
specific information for determining TTC class  
2) Anti-cholinesterase inhibiting (Anti-ChE Organophospahate ester or
Carbamate Ester) 3) Cramer Class III 4) Cramer Class II 5) Cramer Class
I

```
CERAPP.Toxtree <- CERAPP_Toxtree_Results[!duplicated(CERAPP_Toxtree_Results$dsstox_substance_id), ]

CERAPP.Toxtree$Genotoxic <- ifelse(CERAPP.Toxtree$`Structural Alert for genotoxic carcinogenicity` == "YES" | CERAPP.Toxtree$`Structural Alert for S. typhimurium  mutagenicity` == "YES", "YES", "NO")

CERAPP.Toxtree$Anti_ChE <- ifelse(CERAPP.Toxtree$FG52_2 == "YES" | CERAPP.Toxtree$FG81_2 == "YES", "YES", "NO")

CERAPP.Toxtree$Exposure.Total.Upper95 <- CERAPP_HeuristicExposure$Total.Upper95[match(CERAPP.Toxtree$dsstox_substance_id,CERAPP_HeuristicExposure$DSSTox_Substance_Id)]
CERAPP.Toxtree$Exposure.Total.Median <- CERAPP_HeuristicExposure$Total.Median[match(CERAPP.Toxtree$dsstox_substance_id,CERAPP_HeuristicExposure$DSSTox_Substance_Id)]

CERAPP.Toxtree$oed.AC50p5 <-  m.tmp$oed.AC50p5[match(CERAPP.Toxtree$casrn,m.tmp$chemcas)]
CERAPP.Toxtree$oed.AC50p50 <-  m.tmp$oed.AC50p50[match(CERAPP.Toxtree$casrn,m.tmp$chemcas)]
CERAPP.Toxtree$oed.AC50p95 <-  m.tmp$oed.AC50p95[match(CERAPP.Toxtree$casrn,m.tmp$chemcas)]

# CERAPP.Toxtree$NOAEL <- as.numeric(TRICHIP_IRIS_NOAEL$NOAEL)[match(CERAPP.Toxtree$casrn, TRICHIP_IRIS_NOAEL$casrn)]
CERAPP.Toxtree$NOAEL <- as.numeric(toxvaldb.indivchem$toxval_numeric)[match(CERAPP.Toxtree$casrn, toxvaldb.indivchem$casrn)]

CERAPP.Toxtree <- CERAPP.Toxtree[-which(is.na(CERAPP.Toxtree$Exposure.Total.Upper95) == TRUE),]

CERAPP.Toxtree$preferred_name <- CERAPP_HeuristicExposure$PREFERRED_NAME[match(CERAPP.Toxtree$casrn, CERAPP_HeuristicExposure$Substance_CASRN)]

#Separate out genotox analysis as gCERAPP.Toxtree
gCERAPP.Toxtree <- CERAPP.Toxtree

#Original
cerapp_ttc_vector <- c(1:nrow(CERAPP.Toxtree))
for(i in 1:nrow(CERAPP.Toxtree)){
if(CERAPP.Toxtree$Anti_ChE[i] == "YES" & !is.na(CERAPP.Toxtree$Anti_ChE[i])){
  cerapp_ttc_vector[i] <- 0.3
} else if (CERAPP.Toxtree$`Cramer rules, with extensions`[i] == "High (Class III)" & !is.na(CERAPP.Toxtree$`Cramer rules, with extensions`[i])){
  cerapp_ttc_vector[i] <- 1.5
} else if (CERAPP.Toxtree$`Cramer rules, with extensions`[i] == "Intermediate (Class II)"& !is.na(CERAPP.Toxtree$`Cramer rules, with extensions`[i])){
  cerapp_ttc_vector[i] <- 9
} else if (CERAPP.Toxtree$`Cramer rules, with extensions`[i] == "Low (Class I)"& !is.na(CERAPP.Toxtree$`Cramer rules, with extensions`[i])){
  cerapp_ttc_vector[i] <- 30
} else {
  cerapp_ttc_vector[i] <- NA
}
}
CERAPP.Toxtree$TTC_ug.kg.d <- cerapp_ttc_vector

CERAPP.Toxtree$TTC_Class <- ifelse(CERAPP.Toxtree$TTC_ug.kg.d == 0.0025, "Genotoxic", ifelse(CERAPP.Toxtree$TTC_ug.kg.d == 0.3, "Anti-cholinesterase", ifelse(CERAPP.Toxtree$TTC_ug.kg.d == 1.5, "Cramer Class III", ifelse(CERAPP.Toxtree$TTC_ug.kg.d == 9, "Cramer Class II", ifelse(CERAPP.Toxtree$TTC_ug.kg.d == 30, "Cramer Class I",NA)))))

CERAPP.Toxtree$TTC_MoE <- (0.001*CERAPP.Toxtree$TTC_ug.kg.d)/CERAPP.Toxtree$Exposure.Total.Upper95 #divide by 1000 to get mg/kg/d, already safety factor 100 here
CERAPP.Toxtree$OED_MoE <- (CERAPP.Toxtree$oed.AC50p5)/CERAPP.Toxtree$Exposure.Total.Upper95
CERAPP.Toxtree$NOAEL_MoE <- (CERAPP.Toxtree$NOAEL)/CERAPP.Toxtree$Exposure.Total.Upper95 #divide by safety factor of 100

CERAPP.Toxtree <- CERAPP.Toxtree[-which(is.na(CERAPP.Toxtree$TTC_MoE)==TRUE),]

#Genotox
gcerapp_ttc_vector <- c(1:nrow(gCERAPP.Toxtree))
for(i in 1:nrow(gCERAPP.Toxtree)){
if (gCERAPP.Toxtree$Genotoxic[i] == "YES" & !is.na(gCERAPP.Toxtree$Genotoxic[i])){
  gcerapp_ttc_vector[i] <- 0.0025
} else if(gCERAPP.Toxtree$Anti_ChE[i] == "YES" & !is.na(gCERAPP.Toxtree$Anti_ChE[i])){
  gcerapp_ttc_vector[i] <- 0.3
} else if (gCERAPP.Toxtree$`Cramer rules, with extensions`[i] == "High (Class III)" & !is.na(gCERAPP.Toxtree$`Cramer rules, with extensions`[i])){
  gcerapp_ttc_vector[i] <- 1.5
} else if (gCERAPP.Toxtree$`Cramer rules, with extensions`[i] == "Intermediate (Class II)"& !is.na(gCERAPP.Toxtree$`Cramer rules, with extensions`[i])){
  gcerapp_ttc_vector[i] <- 9
} else if (gCERAPP.Toxtree$`Cramer rules, with extensions`[i] == "Low (Class I)"& !is.na(gCERAPP.Toxtree$`Cramer rules, with extensions`[i])){
  gcerapp_ttc_vector[i] <- 30
} else {
  gcerapp_ttc_vector[i] <- NA
}
}
gCERAPP.Toxtree$TTC_ug.kg.d <- gcerapp_ttc_vector

gCERAPP.Toxtree$TTC_Class <- ifelse(gCERAPP.Toxtree$TTC_ug.kg.d == 0.0025, "Genotoxic", ifelse(gCERAPP.Toxtree$TTC_ug.kg.d == 0.3, "Anti-cholinesterase", ifelse(gCERAPP.Toxtree$TTC_ug.kg.d == 1.5, "Cramer Class III", ifelse(gCERAPP.Toxtree$TTC_ug.kg.d == 9, "Cramer Class II", ifelse(gCERAPP.Toxtree$TTC_ug.kg.d == 30, "Cramer Class I",NA)))))

gCERAPP.Toxtree$TTC_MoE <- (0.001*gCERAPP.Toxtree$TTC_ug.kg.d)/gCERAPP.Toxtree$Exposure.Total.Upper95 #divide by 1000 to get mg/kg/d, already safety factor 100 here
gCERAPP.Toxtree$OED_MoE <- (gCERAPP.Toxtree$oed.AC50p5)/gCERAPP.Toxtree$Exposure.Total.Upper95
gCERAPP.Toxtree$NOAEL_MoE <- (gCERAPP.Toxtree$NOAEL)/gCERAPP.Toxtree$Exposure.Total.Upper95 #divide by safety factor of 100

gCERAPP.Toxtree <- gCERAPP.Toxtree[-which(is.na(gCERAPP.Toxtree$TTC_MoE)==TRUE),]


dim(CERAPP.Toxtree) #45015
```

```
## [1] 45015    48
```

```
dim(gCERAPP.Toxtree) #45015
```

```
## [1] 45038    48
```

# CHEMICAL FILTER CHECK

## Starting List of Chemicals: httk

```
print(paste0(nrow(chem.dt), " chemicals in httk dataset"))
```

```
## [1] "998 chemicals in httk dataset"
```

## TTC List of Chemicals: Toxtree + SEEM3

```
toxtree.count <- merge(HTTK_Toxtree,httk_chems[!duplicated(httk_chems$INPUT)], by.x = c("CAS"),by.y = c("INPUT"))
print(paste0(length(unique(toxtree.count$PREFERRED_NAME)), " chemicals in ToxTree (httk chemicals) dataset"))
```

```
## [1] "966 chemicals in ToxTree (httk chemicals) dataset"
```

```
print(paste0(nrow(seem3db.pos), " chemicals in SEEM3 dataset"))
```

```
## [1] "750 chemicals in SEEM3 dataset"
```

```
db1 <- merge(seem3db.pos,toxtree.count,by = "CAS")
db1 <- db1[!duplicated(dsstox_substance_id),]
print(paste0(nrow(db1), " chemicals in TTC dataset"))
```

```
## [1] "745 chemicals in TTC dataset"
```

## OED List of Chemicals: ToxCast + SEEM3 (+ httk)

```
print(paste0(length(unique(m.tmp$chemcas)), " chemicals in ToxCast (httk chemicals) dataset"))
```

```
## [1] "915 chemicals in ToxCast (httk chemicals) dataset"
```

```
print(paste0(nrow(seem3db.pos), " chemicals in SEEM3 dataset"))
```

```
## [1] "750 chemicals in SEEM3 dataset"
```

```
db2 <- merge(seem3db.pos,m.tmp[!duplicated(m.tmp$chemcas),],by.x = "CAS", by.y = "chemcas")
db2 <- db2[!duplicated(dsstox_substance_id),]
print(paste0(nrow(db2), " chemicals in OED dataset"))
```

```
## [1] "714 chemicals in OED dataset"
```

## NOAEL List of Chemicals: ToxValDB + SEEM3

```
thdb <- toxvaldb.indivchem[match(chem.dt$chemcas,toxvaldb.indivchem$casrn),]
thdb <- thdb[!is.na(thdb$casrn),]
print(paste0(nrow(thdb), " chemicals in ToxValDB (httk chemicals) dataset"))
```

```
## [1] "592 chemicals in ToxValDB (httk chemicals) dataset"
```

```
print(paste0(nrow(seem3db.pos), " chemicals in SEEM3 dataset"))
```

```
## [1] "750 chemicals in SEEM3 dataset"
```

```
db3 <- merge(seem3db.pos,toxvaldb.indivchem,by.x = "CAS", by.y = "casrn")
db3 <- db3[!duplicated(dsstox_substance_id),]
print(paste0(nrow(db3), " chemicals in NOAEL dataset"))
```

```
## [1] "538 chemicals in NOAEL dataset"
```

## Overlap 1: TTC + OED

```
db1_2 <- merge(db2,db1,by = "dsstox_substance_id")
print(paste0(nrow(db1_2), " chemicals in TTC/OED overlap dataset"))
```

```
## [1] "709 chemicals in TTC/OED overlap dataset"
```

## Overlap 2: TTC + OED + NOAEL

```
db1_2_3 <- merge(db3,db1_2,by = "dsstox_substance_id")
print(paste0(nrow(db1_2_3), " chemicals in TTC/OED/NOAEL dataset"))
```

```
## [1] "522 chemicals in TTC/OED/NOAEL dataset"
```

# IN-TEXT RESULTS

## Section 3.1

How many chemicals in the httk chemical dataset have TTCs, OEDs, and
Exposure estimates?

```
# length(which(get_cheminfo() %in% tc.dt$casn) == TRUE) #935
HTTK.Toxtree <- HTTK.Toxtree[!duplicated(DTXSID)] #2 chemicals had duplicate CAS numbers that were pulled in by the Chemicals Dashboard
nrow(HTTK.Toxtree) #709
```

```
## [1] 709
```

How many chemicals in the httk dataset were deemed inappropriate
based on Kroes Decision Tree method?

```
length(which(HTTK.Toxtree$`Kroes TTC decision tree` == "Risk assessment requires compound-specific toxicity data")) #53 no exposure estimate, 130 exposure estimate
```

```
## [1] 130
```

```
length(which(HTTK.Toxtree$`Kroes TTC decision tree` == "Risk assessment requires compound-specific toxicity data"))/nrow(HTTK.Toxtree)*100 #7.5%
```

```
## [1] 18.33568
```

### Figure 2: Box and whisker plots of OEDs and SEEM Exposures by TTC Class

```
cbPalette <- c("#E69F00", "#56B4E9","#000000","#009E73", "#F0E442", "#0072B2", "#D55E00", "#CC79A7") #Setting a colorblind friendly palette
exposureData <- data.frame(value = HTTK.Toxtree$Exposure.Total.Upper95,
                          class= HTTK.Toxtree$TTC_Class,
                          variable = rep("Exposure", nrow(HTTK.Toxtree)),
                          stringsAsFactors = F )
OEDData <- data.frame(value = HTTK.Toxtree$oed.AC50p5,
                          class= HTTK.Toxtree$TTC_Class,
                          variable = rep("OED", nrow(HTTK.Toxtree)),
                          stringsAsFactors = F )

df<-rbind(exposureData, OEDData)
# df$class <- factor(df$class, levels = c("Genotoxic", "Anti-cholinesterase", "Cramer Class III", "Cramer Class II", "Cramer Class I"))
df$class <- factor(df$class, levels = c("Anti-cholinesterase", "Cramer Class III", "Cramer Class II", "Cramer Class I"))
f_labels <- data.frame(class = factor(c("Anti-cholinesterase", "Cramer Class III", "Cramer Class II", "Cramer Class I"),
                                      levels = c("Anti-cholinesterase", "Cramer Class III", "Cramer Class II", "Cramer Class I")),
                       label = c(paste0("n = ",nrow(df[df$class == "Anti-cholinesterase",])/2),
                                 paste0("n = ",nrow(df[df$class == "Cramer Class III",])/2),
                                 paste0("n = ",nrow(df[df$class == "Cramer Class II",])/2),
                                 paste0("n = ",nrow(df[df$class == "Cramer Class I",])/2)))
head(df)
```

```
##         value               class variable
## 1 0.003575010 Anti-cholinesterase Exposure
## 2 3.163102133    Cramer Class III Exposure
## 3 0.002276418    Cramer Class III Exposure
## 4 0.000845009    Cramer Class III Exposure
## 5 0.000929534    Cramer Class III Exposure
## 6 0.001264670    Cramer Class III Exposure
```

```
#plot
hline.dat<- data.frame(class=factor(levels(df$class),levels = c("Anti-cholinesterase", "Cramer Class III", "Cramer Class II", "Cramer Class I")),
                       # hl = c((0.001*0.0025), (0.001*0.3),(0.001*1.5),(0.001*9), (0.001*30)),
                       hl = c((0.001*0.3),(0.001*1.5),(0.001*9), (0.001*30)),
                       # f= c("#386cb0","#fdb462","#7fc97f","#ef3b2c","#662506" ))
                       f= c("#fdb462","#7fc97f","#ef3b2c","#662506" ))
p<-ggplot(df, aes(variable, value)) + 
  geom_boxplot(aes(color=variable),outlier.size = 3,lwd=1) + 
  geom_hline(data=hline.dat, aes(yintercept = hl), size=1.5 , color ="black", linetype = 2)+
  geom_richtext(data = f_labels, aes(label = label),x = Inf, y = -Inf, hjust = 1.05, vjust = -0.2, size = 12)+
  scale_y_log10(breaks=c(1E-12,1E-9,1E-6,1E-3,1E0,1E3),labels = trans_format("log10", scales::math_format(10^.x)))+
  facet_grid(~class) +
  scale_color_manual(values = cbPalette)+
  ylab("OEDs and Exposure Estimates (mg/kg/d)\n") + 
  xlab("") +
  theme_bw()+
  theme(legend.title=element_blank(),
        legend.key = element_rect(fill = "transparent", colour = "transparent"),
        #toggle line below white bg with grey gridlines or grey bg with white
        # panel.background = element_blank(), panel.grid.minor =  element_line(color = "gray89"), panel.grid.major =  element_line(color = "gray89"), 
        panel.border =  element_rect(colour = "black", fill=NA, size=.7),
        axis.title.x=element_blank(),
        axis.text.x=element_blank(),
        axis.ticks.x=element_blank(),
        strip.text = element_text(colour = "white",size = 24, face="bold"),
        legend.text = element_text(size = 30, face = 'bold'),
        axis.text.y = element_text(size = 28, face = 'bold', color = 'black'),
        axis.title.y = element_text(size = 30, face = 'bold'))

p
```

```
png(file = paste0(subDir,"/FIGURE_2_boxplot_httk_oedvseem_lines.png"), bg = "transparent",width=18,height=10,units="in",res=300)
g <- ggplot_gtable(ggplot_build(p))
stripr <- which(grepl('strip-t', g$layout$name))
fills <- c("black","black","black","black","black")
# fills <- c("#386cb0","#fdb462","#7fc97f","#ef3b2c","#662506" )
k <- 1
for (i in stripr) {
  j <- which(grepl('strip.background', g$grobs[[i]]$grobs[[1]]$childrenOrder))
  g$grobs[[i]]$grobs[[1]]$children[[j]]$gp$fill <- fills[k]
  k <- k+1
}
grid.newpage();grid.draw(g)


dev.off()
```

```
## png 
##   2
```

### Figure 2b: Box and whisker plots of OEDs and SEEM Exposures by TTC Class (including genotox)

```
cbPalette <- c("#E69F00", "#56B4E9","#000000","#009E73", "#F0E442", "#0072B2", "#D55E00", "#CC79A7") #Setting a colorblind friendly palette
exposureData <- data.frame(value = gHTTK.Toxtree$Exposure.Total.Upper95,
                          class= gHTTK.Toxtree$TTC_Class,
                          variable = rep("Exposure", nrow(gHTTK.Toxtree)),
                          stringsAsFactors = F )
OEDData <- data.frame(value = gHTTK.Toxtree$oed.AC50p5,
                          class= gHTTK.Toxtree$TTC_Class,
                          variable = rep("OED", nrow(gHTTK.Toxtree)),
                          stringsAsFactors = F )

df<-rbind(exposureData, OEDData)
df$class <- factor(df$class, levels = c("Genotoxic", "Anti-cholinesterase", "Cramer Class III", "Cramer Class II", "Cramer Class I"))
f_labels <- data.frame(class = factor(c("Genotoxic","Anti-cholinesterase", "Cramer Class III", "Cramer Class II", "Cramer Class I"),
                                      levels = c("Genotoxic","Anti-cholinesterase", "Cramer Class III", "Cramer Class II", "Cramer Class I")),
                       label = c(paste0("n = ",nrow(df[df$class == "Genotoxic",])/2),
                                 paste0("n = ",nrow(df[df$class == "Anti-cholinesterase",])/2),
                                 paste0("n = ",nrow(df[df$class == "Cramer Class III",])/2),
                                 paste0("n = ",nrow(df[df$class == "Cramer Class II",])/2),
                                 paste0("n = ",nrow(df[df$class == "Cramer Class I",])/2)))
head(df)
```

```
##         value            class variable
## 1 0.003575010        Genotoxic Exposure
## 2 3.163102133        Genotoxic Exposure
## 3 0.002276418 Cramer Class III Exposure
## 4 0.000845009 Cramer Class III Exposure
## 5 0.000929534        Genotoxic Exposure
## 6 0.001264670        Genotoxic Exposure
```

```
#plot
hline.dat<- data.frame(class=factor(levels(df$class),levels = c("Genotoxic","Anti-cholinesterase", "Cramer Class III", "Cramer Class II", "Cramer Class I")),
                       hl = c((0.001*0.0025), (0.001*0.3),(0.001*1.5),(0.001*9), (0.001*30)),
                       f= c("#386cb0","#fdb462","#7fc97f","#ef3b2c","#662506" ))
p<-ggplot(df, aes(variable, value)) + 
  geom_boxplot(aes(color=variable),outlier.size = 3,lwd=1) + 
  geom_hline(data=hline.dat, aes(yintercept = hl), size=1.5 , color ="black", linetype = 2)+
  geom_richtext(data = f_labels, aes(label = label),x = Inf, y = -Inf, hjust = 1.05, vjust = -0.2, size = 12)+
  scale_y_log10(breaks=c(1E-12,1E-9,1E-6,1E-3,1E0,1E3),labels = trans_format("log10", scales::math_format(10^.x)))+
  facet_grid(~class) +
  scale_color_manual(values = cbPalette)+
  ylab("OEDs and Exposure Estimates (mg/kg/d)\n") + 
  xlab("") +
  theme_bw()+
  theme(legend.title=element_blank(),
        legend.key = element_rect(fill = "transparent", colour = "transparent"),
        #toggle line below white bg with grey gridlines or grey bg with white
        # panel.background = element_blank(), panel.grid.minor =  element_line(color = "gray89"), panel.grid.major =  element_line(color = "gray89"), 
        panel.border =  element_rect(colour = "black", fill=NA, size=.7),
        axis.title.x=element_blank(),
        axis.text.x=element_blank(),
        axis.ticks.x=element_blank(),
        strip.text = element_text(colour = "white",size = 24, face="bold"),
        legend.text = element_text(size = 30, face = 'bold'),
        axis.text.y = element_text(size = 28, face = 'bold', color = 'black'),
        axis.title.y = element_text(size = 30, face = 'bold'))

p
```

```
png(file = paste0(subDir,"/FIGURE_2b_boxplot_httk_oedvseem_lines_genotox.png"), bg = "transparent",width=20.5,height=10,units="in",res=300)
g <- ggplot_gtable(ggplot_build(p))
stripr <- which(grepl('strip-t', g$layout$name))
fills <- c("black","black","black","black","black")
# fills <- c("#386cb0","#fdb462","#7fc97f","#ef3b2c","#662506" )
k <- 1
for (i in stripr) {
  j <- which(grepl('strip.background', g$grobs[[i]]$grobs[[1]]$childrenOrder))
  g$grobs[[i]]$grobs[[1]]$children[[j]]$gp$fill <- fills[k]
  k <- k+1
}
grid.newpage();grid.draw(g)


dev.off()
```

```
## png 
##   2
```

### Figure 2 Redone: Empirical Cumulative Density Function plots of OEDs and SEEM Exposures by TTC Class

```
cbPalette <- c("#E69F00", "#56B4E9","#000000","#009E73", "#F0E442", "#0072B2", "#D55E00", "#CC79A7") #Setting a colorblind friendly palette
exposureData <- data.frame(value = HTTK.Toxtree$Exposure.Total.Upper95,
                          class= HTTK.Toxtree$TTC_Class,
                          variable = rep("Exposure", nrow(HTTK.Toxtree)),
                          stringsAsFactors = F )
OEDData <- data.frame(value = HTTK.Toxtree$oed.AC50p5,
                          class= HTTK.Toxtree$TTC_Class,
                          variable = rep("OED", nrow(HTTK.Toxtree)),
                          stringsAsFactors = F )

df<-rbind(exposureData, OEDData)
# df$class <- factor(df$class, levels = c("Genotoxic", "Anti-cholinesterase", "Cramer Class III", "Cramer Class II", "Cramer Class I"))
df$class <- factor(df$class, levels = c("Anti-cholinesterase", "Cramer Class III", "Cramer Class II", "Cramer Class I"))
f_labels <- data.frame(class = factor(c("Anti-cholinesterase", "Cramer Class III", "Cramer Class II", "Cramer Class I"),
                                      levels = c("Anti-cholinesterase", "Cramer Class III", "Cramer Class II", "Cramer Class I")),
                       label = c(paste0("n = ",nrow(df[df$class == "Anti-cholinesterase",])/2),
                                 paste0("n = ",nrow(df[df$class == "Cramer Class III",])/2),
                                 paste0("n = ",nrow(df[df$class == "Cramer Class II",])/2),
                                 paste0("n = ",nrow(df[df$class == "Cramer Class I",])/2)))
head(df)
```

```
##         value               class variable
## 1 0.003575010 Anti-cholinesterase Exposure
## 2 3.163102133    Cramer Class III Exposure
## 3 0.002276418    Cramer Class III Exposure
## 4 0.000845009    Cramer Class III Exposure
## 5 0.000929534    Cramer Class III Exposure
## 6 0.001264670    Cramer Class III Exposure
```

```
#plot
hline.dat<- data.frame(class=factor(levels(df$class),levels = c("Anti-cholinesterase", "Cramer Class III", "Cramer Class II", "Cramer Class I")),
                       # hl = c((0.001*0.0025), (0.001*0.3),(0.001*1.5),(0.001*9), (0.001*30)),
                       hl = c((0.001*0.3),(0.001*1.5),(0.001*9), (0.001*30)),
                       # f= c("#386cb0","#fdb462","#7fc97f","#ef3b2c","#662506" ))
                       f= c("#fdb462","#7fc97f","#ef3b2c","#662506" ))
p<-ggplot(df, aes(value)) + 
  stat_ecdf(aes(color=variable),geom = 'step',size = 1.5) + 
  geom_vline(data=hline.dat, aes(xintercept = hl), size=1.5 , color ="black", linetype = 2)+
  geom_richtext(data = f_labels, aes(label = label),x = -Inf, y = Inf, hjust = -0.05, vjust = 1.15, size = 12)+
  facet_grid(~class,scales = "free_x") +
  scale_x_log10(labels = trans_format("log10", scales::math_format(10^.x)))+
  scale_color_manual(values = cbPalette)+
  ylab("Cumulative Density\n") + 
  xlab("\nOEDs and Exposure Estimates (mg/kg/d)") +
  theme_bw()+
  theme(legend.title=element_blank(),
        legend.key = element_rect(fill = "transparent", colour = "transparent"),
        #toggle line below white bg with grey gridlines or grey bg with white
        # panel.background = element_blank(), panel.grid.minor =  element_line(color = "gray89"), panel.grid.major =  element_line(color = "gray89"), 
        panel.border =  element_rect(colour = "black", fill=NA, size=.7),
        # axis.title.x=element_blank(),
        # axis.text.x=element_blank(),
        # axis.ticks.x=element_blank(),
        strip.text = element_text(colour = "white",size = 24, face="bold"),
        legend.text = element_text(size = 30, face = 'bold'),
        axis.text.x = element_text(size = 28, face = 'bold', color = 'black'),
        axis.title.x = element_text(size = 30, face = 'bold'),
        axis.text.y = element_text(size = 28, face = 'bold', color = 'black'),
        axis.title.y = element_text(size = 30, face = 'bold'))

print(p)
```

```
# png(file = paste0(subDir,"/FIGURE_2_ecdf_httk_oedvseem_lines.png"), bg = "transparent",width=22,height=10,units="in",res=300)
# g <- ggplot_gtable(ggplot_build(p))
# stripr <- which(grepl('strip-t', g$layout$name))
# fills <- c("black","black","black","black","black")
# # fills <- c("#386cb0","#fdb462","#7fc97f","#ef3b2c","#662506" )
# k <- 1
# for (i in stripr) {
#   j <- which(grepl('strip.background', g$grobs[[i]]$grobs[[1]]$childrenOrder))
#   g$grobs[[i]]$grobs[[1]]$children[[j]]$gp$fill <- fills[k]
#   k <- k+1
# }
# grid.newpage();grid.draw(g)


dev.off()
```

```
## null device 
##           1
```

Calculate stats for the group

```
#https://miningthedetails.com/blog/r/non-parametric-tests/
#Are the medians different?
print("Comparing Medians")
```

```
## [1] "Comparing Medians"
```

```
accomp <- wilcox.test(df$value[df$class == "Anti-cholinesterase" & df$variable == "Exposure"],
       df$value[df$class == "Anti-cholinesterase" & df$variable == "OED"])
c3comp <- wilcox.test(df$value[df$class == "Cramer Class III" & df$variable == "Exposure"],
       df$value[df$class == "Cramer Class III" & df$variable == "OED"])
c2comp <- wilcox.test(df$value[df$class == "Cramer Class II" & df$variable == "Exposure"],
       df$value[df$class == "Cramer Class II" & df$variable == "OED"])
c1comp <- wilcox.test(df$value[df$class == "Cramer Class I" & df$variable == "Exposure"],
       df$value[df$class == "Cramer Class I" & df$variable == "OED"])
print(c(accomp$p.value,c3comp$p.value,c2comp$p.value,c1comp$p.value))
```

```
## [1] 4.050885e-16 1.816092e-79 8.534283e-01 2.964622e-07
```

```
#Are the distributions different?
print("Comparing Distrbutions")
```

```
## [1] "Comparing Distrbutions"
```

```
accomp <- ks.test(df$value[df$class == "Anti-cholinesterase" & df$variable == "Exposure"],
       df$value[df$class == "Anti-cholinesterase" & df$variable == "OED"])
c3comp <- ks.test(df$value[df$class == "Cramer Class III" & df$variable == "Exposure"],
       df$value[df$class == "Cramer Class III" & df$variable == "OED"])
c2comp <- ks.test(df$value[df$class == "Cramer Class II" & df$variable == "Exposure"],
       df$value[df$class == "Cramer Class II" & df$variable == "OED"])
c1comp <- ks.test(df$value[df$class == "Cramer Class I" & df$variable == "Exposure"],
       df$value[df$class == "Cramer Class I" & df$variable == "OED"])
print(c(accomp$p.value,c3comp$p.value,c2comp$p.value,c1comp$p.value))
```

```
## [1] 6.505907e-14 0.000000e+00 7.869298e-01 7.126555e-08
```

```
#Statistical test options: t.test(), ks.test(), wilcox.test(), https://en.wikipedia.org/wiki/Kolmogorov%E2%80%93Smirnov_test
```

How many HTTK chemicals have an OED based MoE less than 1

```
h.moe.oed.below.1 <-length(which(HTTK.Toxtree$OED_MoE < 1)) 
h.moe.oed.below.1 #113
```

```
## [1] 113
```

```
pct.h.moe.oed.below.1 <- 100*(h.moe.oed.below.1/nrow(HTTK.Toxtree)) #2.3%, now 3.6, still 3.6% (no genotox)
pct.h.moe.oed.below.1 #15.9%
```

```
## [1] 15.93794
```

How many HTTK chemicals have a TTC based MoE less than 1?

```
h.moe.ttc.below.1 <-length(which(HTTK.Toxtree$TTC_MoE < 1)) 
h.moe.ttc.below.1 #250
```

```
## [1] 250
```

```
pct.h.moe.below.1 <- 100*(h.moe.ttc.below.1/nrow(HTTK.Toxtree)) 
pct.h.moe.below.1 #35.3%
```

```
## [1] 35.26093
```

### Table 2

```
#How many (and what %) chemicals in the httk chemical dataset are grouped in to each of the 4 TTC categories?
h.antiche <-length(which(HTTK.Toxtree$TTC_Class == "Anti-cholinesterase")) #34
h.classIII <- length(which(HTTK.Toxtree$TTC_Class == "Cramer Class III")) #525
h.classII <- length(which(HTTK.Toxtree$TTC_Class == "Cramer Class II")) #10
h.classI <- length(which(HTTK.Toxtree$TTC_Class == "Cramer Class I")) #140

h.total <- sum(h.antiche,h.classIII, h.classII, h.classI) #709

pct.h.antiche <- round(100*(h.antiche/h.total),1) #4.8%
pct.h.classIII <- round(100*(h.classIII/h.total),1) #74.0%
pct.h.classII <- round(100*(h.classII/h.total),1) #1.4%
pct.h.classI<- round(100*(h.classI/h.total),1) #19.7%


#How many (and what % of a given category) chemicals with OED MoE < 1 in the httk chemical dataset are grouped in to each of the 4 TTC categories?
httk.oed.moe.less.one <- HTTK.Toxtree[which(HTTK.Toxtree$OED_MoE <1),]

homloa <- length(which(httk.oed.moe.less.one$TTC_Class == "Anti-cholinesterase")) #1
homloa.per <- round(homloa/h.antiche * 100,1) #2.9% of ACHEi chemicals

homlo3 <- length(which(httk.oed.moe.less.one$TTC_Class == "Cramer Class III")) #69
homlo3.per <- round(homlo3/h.classIII * 100,1) #13.1% of CCIII chemicals

homlo2 <- length(which(httk.oed.moe.less.one$TTC_Class == "Cramer Class II")) #4
homlo2.per <- round(homlo2/h.classII * 100,1) #40% of CCII chemicals

homlo1 <- length(which(httk.oed.moe.less.one$TTC_Class == "Cramer Class I")) #39
homlo1.per <- round(homlo1/h.classI * 100,1) #27.9% of CCI chemicals

homlotot <- homloa + homlo3 + homlo2 + homlo1 #113
homlotot.per <- round(homlotot/h.total * 100,1) #15.9%

#How many (and what % of a given category) chemicals with TTC MoE < 1 in the httk chemical dataset are grouped in to each of the 4 TTC categories?
httk.ttc.moe.less.one <- HTTK.Toxtree[which(HTTK.Toxtree$TTC_MoE <1),]

htmloa <- length(which(httk.ttc.moe.less.one$TTC_Class == "Anti-cholinesterase")) #16
htmloa.per <- round(htmloa/h.antiche * 100,1) #47.1% of ACHEi chemicals

htmlo3 <- length(which(httk.ttc.moe.less.one$TTC_Class == "Cramer Class III")) #168
htmlo3.per <- round(htmlo3/h.classIII*100,1) #32.0% of CCIII chemicals

htmlo2 <- length(which(httk.ttc.moe.less.one$TTC_Class == "Cramer Class II")) #6
htmlo2.per <- round(htmlo2/h.classII*100,1) #60% of CCII chemicals

htmlo1 <- length(which(httk.ttc.moe.less.one$TTC_Class == "Cramer Class I")) #60
htmlo1.per <- round(htmlo1/h.classI*100,1) #42.9% of CCI chemicals

htmlotot <- htmloa + htmlo3 + htmlo2 + htmlo1 #250
htmlotot.per <- round(htmlotot/h.total * 100,1) #35.3%

t2 <- data.table("TTC Class and MoE Results" = c("Anti-cholinesterase Inhibitors",
                                                 "Cramer Class III",
                                                 "Cramer Class II",
                                                 "Cramer Class I",
                                                 "Total"),
                 "Number of Compounds (% of Compounds)" = c(paste0(h.antiche," (",pct.h.antiche,"%)"),
                                                            paste0(h.classIII," (",pct.h.classIII,"%)"),
                                                            paste0(h.classII," (",pct.h.classII,"%)"),
                                                            paste0(h.classI," (",pct.h.classI,"%)"),
                                                            paste0(h.total," (100%)")),
                 "OED-based MoEs < 1 (% of Compounds in Class)" = c(paste0(homloa," (",homloa.per,"%)"),
                                                            paste0(homlo3," (",homlo3.per,"%)"),
                                                            paste0(homlo2," (",homlo2.per,"%)"),
                                                            paste0(homlo1," (",homlo1.per,"%)"),
                                                            paste0(homlotot," (",homlotot.per,"%)")),
                 "TTC-based MoEs < 1 (% of Compounds in Class)" = c(paste0(htmloa," (",htmloa.per,"%)"),
                                                            paste0(htmlo3," (",htmlo3.per,"%)"),
                                                            paste0(htmlo2," (",htmlo2.per,"%)"),
                                                            paste0(htmlo1," (",htmlo1.per,"%)"),
                                                            paste0(htmlotot," (",htmlotot.per,"%)")))
t2
```

```
##         TTC Class and MoE Results Number of Compounds (% of Compounds)
## 1: Anti-cholinesterase Inhibitors                            34 (4.8%)
## 2:               Cramer Class III                            525 (74%)
## 3:                Cramer Class II                            10 (1.4%)
## 4:                 Cramer Class I                          140 (19.7%)
## 5:                          Total                           709 (100%)
##    OED-based MoEs < 1 (% of Compounds in Class)
## 1:                                     1 (2.9%)
## 2:                                   69 (13.1%)
## 3:                                      4 (40%)
## 4:                                   39 (27.9%)
## 5:                                  113 (15.9%)
##    TTC-based MoEs < 1 (% of Compounds in Class)
## 1:                                   16 (47.1%)
## 2:                                    168 (32%)
## 3:                                      6 (60%)
## 4:                                   60 (42.9%)
## 5:                                  250 (35.3%)
```

In-text callouts for Table 2

```
c(homlotot,homlotot.per) #113, 15.9%
```

```
## [1] 113.0  15.9
```

```
c(htmlotot,htmlotot.per) #250, 35.3%
```

```
## [1] 250.0  35.3
```

What are median margin of exposures per class for OED-based MoEs?

```
aggregate(HTTK.Toxtree[, "OED_MoE"], list(HTTK.Toxtree$TTC_Class), median)
```

```
##               Group.1     OED_MoE
## 1 Anti-cholinesterase 2297.458865
## 2      Cramer Class I    8.060190
## 3     Cramer Class II    5.624805
## 4    Cramer Class III   75.689352
```

For TTC-based MoEs?

```
aggregate(HTTK.Toxtree[, "TTC_MoE"], list(HTTK.Toxtree$TTC_Class), median)
```

```
##               Group.1  TTC_MoE
## 1 Anti-cholinesterase 1.072574
## 2      Cramer Class I 3.121397
## 3     Cramer Class II 0.327550
## 4    Cramer Class III 2.939286
```

### Table 3: What are the top 25 HTTK chemicals are prioritized by OED-based MoE? TTC-based?

```
top.25.httk.oed.moe<- setorder(HTTK.Toxtree,OED_MoE)[c(1:25),]
top.25.httk.ttc.moe<- setorder(HTTK.Toxtree,TTC_MoE)[c(1:25),]
table(top.25.httk.oed.moe$PREFERRED_NAME %in% top.25.httk.ttc.moe$PREFERRED_NAME)[2]
```

```
## TRUE 
##   10
```

```
tab3 <- tibble("Ranking (OED-Based)" = 1:25,
       "Chemical (OED-Based)" = top.25.httk.oed.moe$PREFERRED_NAME,
       "Ranking (TTC-Based)" = 1:25,
       "Chemical (TTC-Based)" = top.25.httk.ttc.moe$PREFERRED_NAME)
tab3
```

```
## # A tibble: 25 × 4
##    `Ranking (OED-Based)` `Chemical (OED-Based)`                  Ranki…¹ Chemi…²
##                    <int> <chr>                                     <int> <chr>  
##  1                     1 Saccharin                                     1 Nitrap…
##  2                     2 Octane                                        2 Caprol…
##  3                     3 Coumarin                                      3 2-Merc…
##  4                     4 Sodium 2-mercaptobenzothiolate                4 Oleyl …
##  5                     5 Diallyl phthalate                             5 Bis(2-…
##  6                     6 Dibutyl adipate                               6 Acrylo…
##  7                     7 2,6-Di-tert-butyl-4-[(dimethylamino)me…       7 Cyazof…
##  8                     8 Nicotinic acid                                8 1-Benz…
##  9                     9 Methanol                                      9 1,2-Di…
## 10                    10 Estrone                                      10 Nicoti…
## # … with 15 more rows, and abbreviated variable names ¹​`Ranking (TTC-Based)`,
## #   ²​`Chemical (TTC-Based)`
## # ℹ Use `print(n = ...)` to see more rows
```

```
# write.csv(tab3,paste0(subDir,"/Table_3.csv"),row.names = F)
```

Where is the overlap in Table 3?

```
top.25.httk.oed.moe$PREFERRED_NAME[which(top.25.httk.oed.moe$PREFERRED_NAM %in% top.25.httk.ttc.moe$PREFERRED_NAME)] #10
```

```
##  [1] "Saccharin"                                  
##  [2] "Sodium 2-mercaptobenzothiolate"             
##  [3] "Dibutyl adipate"                            
##  [4] "Nicotinic acid"                             
##  [5] "Methanol"                                   
##  [6] "Caprolactam"                                
##  [7] "1,2-Dimethyl-3-nitrobenzene"                
##  [8] "Nitrapyrin"                                 
##  [9] "2,2'-Methylenebis(ethyl-6-tert-butylphenol)"
## [10] "2-Mercaptobenzimidazole"
```

## Section 3.2

How many chemicals in CERAPP (large) dataset also in small (ToxCast)
dataset?

```
dim(CERAPP.Toxtree) #45015
```

```
## [1] 45015    48
```

```
table(CERAPP.Toxtree$dsstox_substance_id %in% HTTK.Toxtree$DTXSID)[2] #677
```

```
## TRUE 
##  677
```

How many top 25 CERAPP chemicals ranked by TTC are in the top 25 HTTK
chemicals that are ranked by TTC MOE?

```
top.25.cerapp.ttc.moe<- setorder(CERAPP.Toxtree,TTC_MoE)[c(1:25),]
top.25.cerapp.ttc.moe$preferred_name[which(top.25.cerapp.ttc.moe$dsstox_substance_id %in% top.25.httk.ttc.moe$DTXSID)] #1, Nitrapyrin
```

```
## [1] "Nitrapyrin"
```

How many chemicals were deemed inappropriate based on Kroes Decision
Tree method?

```
length(which(CERAPP.Toxtree$`Kroes TTC decision tree` == "Risk assessment requires compound-specific toxicity data")) #5165
```

```
## [1] 5165
```

```
length(which(CERAPP.Toxtree$`Kroes TTC decision tree` == "Risk assessment requires compound-specific toxicity data"))/nrow(CERAPP.Toxtree) * 100 #11.5%
```

```
## [1] 11.47395
```

### Table 4

```
#How many (and what %) chemicals in the CERAPP chemical dataset are grouped in to each of the 4 TTC categories?
c.antiche <-length(which(CERAPP.Toxtree$TTC_Class == "Anti-cholinesterase")) 
c.classIII <- length(which(CERAPP.Toxtree$TTC_Class == "Cramer Class III")) 
c.classII <- length(which(CERAPP.Toxtree$TTC_Class == "Cramer Class II")) 
c.classI <- length(which(CERAPP.Toxtree$TTC_Class == "Cramer Class I")) 
c.total <- sum(c.antiche,c.classIII, c.classII, c.classI)

pct.c.antiche <- round(100*(c.antiche/c.total),1) 
pct.c.classIII <- round(100*(c.classIII/c.total),1) 
pct.c.classII <- round(100*(c.classII/c.total),1) 
pct.c.classI<- round(100*(c.classI/c.total),1) 


#How many (and what % of a given category) chemicals with OED MoE < 1 in the CERAPP chemical dataset are grouped in to each of the 4 TTC categories?
c.oed.moe.less.one <- CERAPP.Toxtree[which(CERAPP.Toxtree$OED_MoE <1),] 

comloa <- length(which(c.oed.moe.less.one$TTC_Class == "Anti-cholinesterase")) 
comloa.per <- round(comloa/c.antiche * 100,1)

comlo3 <- length(which(c.oed.moe.less.one$TTC_Class == "Cramer Class III")) 
comlo3.per <- round(comlo3/c.classIII * 100,1)

comlo2 <- length(which(c.oed.moe.less.one$TTC_Class == "Cramer Class II")) 
comlo2.per <- round(comlo2/c.classII * 100,1)

comlo1 <- length(which(c.oed.moe.less.one$TTC_Class == "Cramer Class I")) 
comlo1.per <- round(comlo1/c.classI * 100,1)

comlotot <- comloa + comlo3 + comlo2 + comlo1
comlotot.per <- round(comlotot/c.total * 100,1)

#How many (and what % of a given category) chemicals with TTC MoE < 1 in the CERAPP chemical dataset are grouped in to each of the 4 TTC categories?
c.ttc.moe.less.one <- CERAPP.Toxtree[which(CERAPP.Toxtree$TTC_MoE <1),] 

ctmloa <- length(which(c.ttc.moe.less.one$TTC_Class == "Anti-cholinesterase")) 
ctmloa.per <- round(ctmloa/c.antiche * 100,1)

ctmlo3 <- length(which(c.ttc.moe.less.one$TTC_Class == "Cramer Class III")) 
ctmlo3.per <- round(ctmlo3/c.classIII * 100,1)

ctmlo2 <- length(which(c.ttc.moe.less.one$TTC_Class == "Cramer Class II")) 
ctmlo2.per <- round(ctmlo2/c.classII * 100,1)

ctmlo1 <- length(which(c.ttc.moe.less.one$TTC_Class == "Cramer Class I")) 
ctmlo1.per <- round(ctmlo1/c.classI * 100,1)

ctmlotot <- ctmloa + ctmlo3 + ctmlo2 + ctmlo1
ctmlotot.per <- round(ctmlotot/c.total * 100,1)

t4 <- data.table("TTC Class and MoE Results" = c("Anti-cholinesterase Inhibitors",
                                                 "Cramer Class III",
                                                 "Cramer Class II",
                                                 "Cramer Class I",
                                                 "Total"),
                 "Number of Compounds (% of Compounds)" = c(paste0(c.antiche," (",pct.c.antiche,"%)"),
                                                            paste0(c.classIII," (",pct.c.classIII,"%)"),
                                                            paste0(c.classII," (",pct.c.classII,"%)"),
                                                            paste0(c.classI," (",pct.c.classI,"%)"),
                                                            paste0(c.total," (100%)")),
                 "OED-based MoEs < 1 (% of Compounds in Class)" = c(paste0(comloa," (",comloa.per,"%)"),
                                                            paste0(comlo3," (",comlo3.per,"%)"),
                                                            paste0(comlo2," (",comlo2.per,"%)"),
                                                            paste0(comlo1," (",comlo1.per,"%)"),
                                                            paste0(comlotot," (",comlotot.per,"%)")),
                 "TTC-based MoEs < 1 (% of Compounds in Class)" = c(paste0(ctmloa," (",ctmloa.per,"%)"),
                                                            paste0(ctmlo3," (",ctmlo3.per,"%)"),
                                                            paste0(ctmlo2," (",ctmlo2.per,"%)"),
                                                            paste0(ctmlo1," (",ctmlo1.per,"%)"),
                                                            paste0(ctmlotot," (",ctmlotot.per,"%)")))
t4
```

```
##         TTC Class and MoE Results Number of Compounds (% of Compounds)
## 1: Anti-cholinesterase Inhibitors                          1506 (3.3%)
## 2:               Cramer Class III                        35772 (79.5%)
## 3:                Cramer Class II                            1346 (3%)
## 4:                 Cramer Class I                         6391 (14.2%)
## 5:                          Total                         45015 (100%)
##    OED-based MoEs < 1 (% of Compounds in Class)
## 1:                                     1 (0.1%)
## 2:                                    84 (0.2%)
## 3:                                     6 (0.4%)
## 4:                                    23 (0.4%)
## 5:                                   114 (0.3%)
##    TTC-based MoEs < 1 (% of Compounds in Class)
## 1:                                  207 (13.7%)
## 2:                                  3158 (8.8%)
## 3:                                   120 (8.9%)
## 4:                                  731 (11.4%)
## 5:                                  4216 (9.4%)
```

### Figure 3: Box and whisker plots of SEEM Exposures by TTC Class for CERAPP

```
df2 <- data.frame(value = CERAPP.Toxtree$TTC_MoE,
                          class= CERAPP.Toxtree$TTC_Class,
                          variable = rep("Exposure", nrow(CERAPP.Toxtree)),
                          stringsAsFactors = F )

df2$class <- factor(df2$class, levels = c("Anti-cholinesterase", "Cramer Class III", "Cramer Class II", "Cramer Class I"))
# head(df2)

#plot
hline.dat<- data.frame(class=factor(levels(df2$class),levels = c("Anti-cholinesterase", "Cramer Class III", "Cramer Class II", "Cramer Class I")),
                       # hl = c((0.001*0.0025),(0.001*0.3),(0.001*1.5),(0.001*9), (0.001*30)),
                       # f= c("#386cb0","#fdb462","#7fc97f","#ef3b2c","#662506" ))
                         hl = c((0.001*0.3),(0.001*1.5),(0.001*9), (0.001*30)),
                         f= c("#fdb462","#7fc97f","#ef3b2c","#662506" ))
p<-ggplot(df2, aes(variable, value)) + 
  geom_boxplot(aes(color=variable),outlier.size = 3,lwd=1) + 
  # geom_hline(data=hline.dat, aes(yintercept = hl), size=1.5 , color ="black")+
  scale_y_log10(breaks=c(1E-6,1E-3,1E0,1E3,1E6,1E9),labels = trans_format("log10", scales::math_format(10^.x)))+
  facet_grid(~class) +
  scale_color_manual(values = c("black", "#00457d"))+
  ylab("TTC-based MoE Estimates (Log Scale)") + 
  xlab("") +
  theme_bw()+
  theme(legend.title=element_blank(),
        legend.key = element_rect(fill = "transparent", colour = "transparent"),
        #toggle line below white bg with grey gridlines or grey bg with white
        # panel.background = element_blank(), panel.grid.minor =  element_line(color = "gray89"), panel.grid.major =  element_line(color = "gray89"), 
        panel.border =  element_rect(colour = "black", fill=NA, size=.7),
        axis.title.x=element_blank(),
        axis.text.x=element_blank(),
        axis.ticks.x=element_blank(),
        strip.text = element_text(colour = "white",size = 24, face="bold"),
        legend.position = "none",
        axis.text.y = element_text(size = 28, face = 'bold', color = 'black'),
        axis.title.y = element_text(size = 30, face = 'bold'))

p
```

```
png(file = paste0(subDir,"/FIGURE_3_boxplot_cerapp_TTC_SEEM_MoE.png"), bg = "transparent",width=18,height=10,units="in",res=300)
g <- ggplot_gtable(ggplot_build(p))
stripr <- which(grepl('strip-t', g$layout$name))
fills <- c("black","black","black","black","black")
# fills <- c("#386cb0","#fdb462","#7fc97f","#ef3b2c","#662506" )
k <- 1
for (i in stripr) {
  j <- which(grepl('strip.background', g$grobs[[i]]$grobs[[1]]$childrenOrder))
  g$grobs[[i]]$grobs[[1]]$children[[j]]$gp$fill <- fills[k]
  k <- k+1
}
grid.newpage();grid.draw(g)


dev.off()
```

```
## png 
##   2
```

### Figure 3b: Box and whisker plots of SEEM Exposures by TTC Class for CERAPP (Genotox)

```
df2 <- data.frame(value = gCERAPP.Toxtree$TTC_MoE,
                          class= gCERAPP.Toxtree$TTC_Class,
                          variable = rep("Exposure", nrow(gCERAPP.Toxtree)),
                          stringsAsFactors = F )

df2$class <- factor(df2$class, levels = c("Genotoxic","Anti-cholinesterase", "Cramer Class III", "Cramer Class II", "Cramer Class I"))
# head(df2)

#plot
hline.dat<- data.frame(class=factor(levels(df2$class),levels = c("Anti-cholinesterase", "Cramer Class III", "Cramer Class II", "Cramer Class I")),
                       hl = c((0.001*0.0025),(0.001*0.3),(0.001*1.5),(0.001*9), (0.001*30)),
                       f= c("#386cb0","#fdb462","#7fc97f","#ef3b2c","#662506" ))

p<-ggplot(df2, aes(variable, value)) + 
  geom_boxplot(aes(color=variable),outlier.size = 3,lwd=1) + 
  # geom_hline(data=hline.dat, aes(yintercept = hl), size=1.5 , color ="black")+
  scale_y_log10(breaks=c(1E-6,1E-3,1E0,1E3,1E6,1E9),labels = trans_format("log10", scales::math_format(10^.x)))+
  facet_grid(~class) +
  scale_color_manual(values = c("black", "#00457d"))+
  ylab("TTC-based MoE Estimates (Log Scale)") + 
  xlab("") +
  theme_bw()+
  theme(legend.title=element_blank(),
        legend.key = element_rect(fill = "transparent", colour = "transparent"),
        #toggle line below white bg with grey gridlines or grey bg with white
        # panel.background = element_blank(), panel.grid.minor =  element_line(color = "gray89"), panel.grid.major =  element_line(color = "gray89"), 
        panel.border =  element_rect(colour = "black", fill=NA, size=.7),
        axis.title.x=element_blank(),
        axis.text.x=element_blank(),
        axis.ticks.x=element_blank(),
        strip.text = element_text(colour = "white",size = 24, face="bold"),
        legend.position = "none",
        axis.text.y = element_text(size = 28, face = 'bold', color = 'black'),
        axis.title.y = element_text(size = 30, face = 'bold'))

p
```

```
png(file = paste0(subDir,"/FIGURE_3b_boxplot_cerapp_TTC_SEEM_MoE_genotox.png"), bg = "transparent",width=18,height=10,units="in",res=300)
g <- ggplot_gtable(ggplot_build(p))
stripr <- which(grepl('strip-t', g$layout$name))
fills <- c("black","black","black","black","black")
# fills <- c("#386cb0","#fdb462","#7fc97f","#ef3b2c","#662506" )
k <- 1
for (i in stripr) {
  j <- which(grepl('strip.background', g$grobs[[i]]$grobs[[1]]$childrenOrder))
  g$grobs[[i]]$grobs[[1]]$children[[j]]$gp$fill <- fills[k]
  k <- k+1
}
grid.newpage();grid.draw(g)


dev.off()
```

```
## png 
##   2
```

Median TTC-MoEs from CERAPP dataset

```
aggregate(CERAPP.Toxtree[, "TTC_MoE"], list(CERAPP.Toxtree$TTC_Class), median)
```

```
##               Group.1   TTC_MoE
## 1 Anti-cholinesterase   3.70142
## 2      Cramer Class I 105.11379
## 3     Cramer Class II  38.70027
## 4    Cramer Class III  16.33987
```

## Section 3.3

```
h.moe.ttc.below.oed <-length(which(HTTK.Toxtree$TTC_MoE < HTTK.Toxtree$OED_MoE)) #590
pct.h.moe.ttc.below.oed <- 100*(h.moe.ttc.below.oed/h.total) #83.2%
print(c(h.moe.ttc.below.oed,pct.h.moe.ttc.below.oed))
```

```
## [1] 590.0000  83.2158
```

### Figure 4:Plot comparing NOAELs to OEDs and TTCs for the first 100

```
httk.MoE.NOAEL <- HTTK.Toxtree[which(is.na(HTTK.Toxtree$NOAEL_MoE)==FALSE),] #522 rows

setorder(httk.MoE.NOAEL,NOAEL_MoE)

httk.MoE.NOAEL$order.NOAEL.MoE <- c(1:nrow(httk.MoE.NOAEL))

httk.MoE.NOAEL.100 <- httk.MoE.NOAEL[c(1:100),]
melt.httk.MoE.NOAEL.100 <- melt(httk.MoE.NOAEL.100[,c(2,5,29,30,31,32)],id=c("INPUT","PREFERRED_NAME","order.NOAEL.MoE"))
p <- ggplot(melt.httk.MoE.NOAEL.100) +
  geom_point(aes(x=reorder(PREFERRED_NAME,order.NOAEL.MoE),y=value, color = variable),size =3)
p <- p + scale_y_log10(limits=c(10E-8,10E5),labels = trans_format("log10", scales::math_format(10^.x))) 
p <- p  + scale_x_discrete(label=function(x) abbreviate(x, minlength=25)) +
  scale_color_manual(name = "Variable",
                     labels = c("TTC MoE","OED MoE","NOAEL MoE"),
                     values = cbPalette)+
  theme_bw()+
  theme(axis.text.x = element_text(size = 8, angle = 60, 
                                   hjust = 1, colour = "grey50"),
        axis.ticks.x = element_line(size=0.01, color = 'grey50'),
        legend.title = element_text(size=rel(1.2)),
        legend.text = element_text(size=rel(1.2))) +
  labs(x='Compound',
       y='TTC-based MoE,\nOED-based MoE, and\nNOAEL-based MoE') #+ scale_color_npg() #+ coord_flip() 

p <- p + theme(axis.text.x=element_text(angle = 90, hjust=1,vjust = 0.5, size=14), axis.title=element_text(size=30,face="bold")) + theme(axis.text.y = element_text( hjust = 1,size=22)) #+ theme(axis.text.x = element_text(hjust = 1,size=20)) 

# p <- p + theme(axis.text=element_text(size=36), axis.title=element_text(size=32,face="bold"))
p <- p + theme(legend.text=element_text(size=20))
p <- p + theme(legend.title = element_text(size=20, face="bold"))

p <- p + theme(legend.justification=c(0.5,0.5), legend.position=c(0.9,0.9), legend.direction = "vertical")
p
```

```
png(file = paste0(subDir,"/FIGURE_4_httk_moe_all.png"), bg = "transparent",width=20,height=12,units="in",res=300)
print(p)
dev.off()
```

```
## png 
##   2
```

Comparing TTC-MoEs to OED-MoEs to NOAEL-MoEs (for chems with all
3)

```
h.moe.oed.below.noael <-length(which(HTTK.Toxtree$OED_MoE < HTTK.Toxtree$NOAEL_MoE)) #457
pct.h.moe.oed.below.noael <- 100*(h.moe.oed.below.noael/length(which(is.na(HTTK.Toxtree$NOAEL_MoE)!=TRUE))) #87.5%
print(c(h.moe.oed.below.noael,pct.h.moe.oed.below.noael))
```

```
## [1] 457.00000  87.54789
```

```
h.moe.ttc.below.noael <-length(which(HTTK.Toxtree$TTC_MoE < HTTK.Toxtree$NOAEL_MoE)) #520
pct.h.moe.ttc.below.noael <- 100*(h.moe.ttc.below.noael/length(which(is.na(HTTK.Toxtree$NOAEL_MoE)!=TRUE))) #99.6%
print(c(h.moe.ttc.below.noael, pct.h.moe.ttc.below.noael))
```

```
## [1] 520.00000  99.61686
```

```
h.moe.ttc.below.oed.part <-length(which(httk.MoE.NOAEL$TTC_MoE < httk.MoE.NOAEL$OED_MoE)) #439
pct.h.moe.ttc.below.oed.part <- 100*(h.moe.ttc.below.oed.part/length(which(is.na(httk.MoE.NOAEL$OED_MoE)!=TRUE))) #84.1%
print(c(h.moe.ttc.below.oed.part, pct.h.moe.ttc.below.oed.part))
```

```
## [1] 439.00000  84.09962
```

### Figure 5: NOAEL-MoEs vs. OED-MoEs or TTC-MoEs

```
httk.overlap <- HTTK.Toxtree[(which(!is.na(HTTK.Toxtree$TTC_MoE) & !is.na(HTTK.Toxtree$OED_MoE) & !is.na(HTTK.Toxtree$NOAEL_MoE))),]
nvo.reg <- lm(log10(NOAEL_MoE)~log10(OED_MoE), data = httk.overlap)
nvt.reg <- lm(log10(NOAEL_MoE)~log10(TTC_MoE), data = httk.overlap)
p4a <- ggplot(data = httk.overlap, aes(x = OED_MoE, y = NOAEL_MoE))+
  geom_point(size = 3)+
  geom_point(data = httk.overlap[httk.overlap$OED_MoE/httk.overlap$NOAEL_MoE < 0.0000001 |
                                  httk.overlap$OED_MoE/httk.overlap$NOAEL_MoE > 10000000 |
                                    (httk.overlap$OED_MoE/httk.overlap$NOAEL_MoE < 1.02 &
                                       httk.overlap$OED_MoE/httk.overlap$NOAEL_MoE > 0.98)],
             size = 3,
             color = 'red')+
  geom_smooth(method = 'lm', se = F, color = "red", formula = y~x)+
  geom_richtext(x = Inf, y = -Inf, label = paste0("r<sup>2</sup> = ",round(summary(nvo.reg)$r.squared,2)), hjust = 1.05, vjust = -0.2, size = 12)+
  geom_text_repel(data = httk.overlap[httk.overlap$OED_MoE/httk.overlap$NOAEL_MoE < 0.0000001 |
                                  httk.overlap$OED_MoE/httk.overlap$NOAEL_MoE > 10000000 |
                                    (httk.overlap$OED_MoE/httk.overlap$NOAEL_MoE < 1.02 &
                                       httk.overlap$OED_MoE/httk.overlap$NOAEL_MoE > 0.98)],
            aes(OED_MoE,
                NOAEL_MoE,
                label = PREFERRED_NAME),
                # label = c("2,4-D",PREFERRED_NAME[2:nrow(httk.overlap[httk.overlap$OED_MoE/httk.overlap$NOAEL_MoE < 0.001 | httk.overlap$OED_MoE/httk.overlap$NOAEL_MoE > 1000|(httk.overlap$OED_MoE/httk.overlap$NOAEL_MoE < 1.1 & httk.overlap$OED_MoE/httk.overlap$NOAEL_MoE > 0.9)])])), #note, this line is a bit unwieldy, but it allows for shortening of "2,4-Dichlorophenoxyacetic acid" for plotting, followed by the remainder of any chemicals desired to be included
            # min.segment.length = 0,
            # box.padding = 12,
            # point.padding = 1,
            max.overlaps = Inf,
            ylim = c(9,10),
            size = 8)+
  scale_x_log10(name = "OED-based Margins of Exposure",
                limits=c(1E-7,1E12),
                labels = trans_format("log10", scales::math_format(10^.x)))+
  scale_y_log10(name = "NOAEL-based Margins of Exposure",
                limits=c(1E-7,1E12),
                labels = trans_format("log10", scales::math_format(10^.x)))+
  geom_abline(slope = 1, intercept = 0, linetype = 2)+
  theme_bw()+
  coord_fixed()+
  labs(tag = "A")+
  theme(axis.text = element_text(size = 25, face = 'bold',color = 'black'),
        axis.title = element_text(size = 30, face = 'bold'),
        plot.tag = element_text(size = 30, face = 'bold'))
p4b <- ggplot(data = httk.overlap, aes(x = TTC_MoE, y = NOAEL_MoE))+
  geom_point(size = 3)+
  geom_point(data = httk.overlap[httk.overlap$TTC_MoE/httk.overlap$NOAEL_MoE < 0.000001 |
                                  httk.overlap$TTC_MoE/httk.overlap$NOAEL_MoE > 1000000|
                                    (httk.overlap$TTC_MoE/httk.overlap$NOAEL_MoE < 1.3 &
                                       httk.overlap$TTC_MoE/httk.overlap$NOAEL_MoE > 0.7)],
             size = 3,
             color = 'red')+
  geom_smooth(method = 'lm', se = F, color = "red", formula = y~x)+
  geom_richtext(x = Inf, y = -Inf, label =  paste0("r<sup>2</sup> = ",round(summary(nvt.reg)$r.squared,2)),hjust = 1.05, vjust = -0.2, size = 12)+
  geom_text_repel(data = httk.overlap[httk.overlap$TTC_MoE/httk.overlap$NOAEL_MoE < 0.000001 |
                                  httk.overlap$TTC_MoE/httk.overlap$NOAEL_MoE > 1000000|
                                    (httk.overlap$TTC_MoE/httk.overlap$NOAEL_MoE < 1.3 &
                                       httk.overlap$TTC_MoE/httk.overlap$NOAEL_MoE > 0.7)],
            aes(TTC_MoE,NOAEL_MoE,label = PREFERRED_NAME),
            # min.segment.length = 0,
            box.padding = 2,
            max.overlaps = Inf,
            ylim = c(9,11),
            size = 8)+
  scale_x_log10(name = "TTC-based Margins of Exposure",
                limits=c(1E-7,1E12),
                labels = trans_format("log10", scales::math_format(10^.x)))+
  scale_y_log10(name = "NOAEL-based Margins of Exposure",
                limits=c(1E-7,1E12),
                labels = trans_format("log10", scales::math_format(10^.x)))+
  geom_abline(slope = 1, intercept = 0, linetype = 2)+
  theme_bw()+
  coord_fixed()+
  labs(tag = "B")+
  theme(axis.text = element_text(size = 25, face = 'bold',color = 'black'),
        axis.title = element_text(size = 30, face = 'bold'),
        plot.tag = element_text(size = 30, face = 'bold'))
print(grid.arrange(p4a,p4b, nrow = 1))
```

```
## TableGrob (1 x 2) "arrange": 2 grobs
##   z     cells    name           grob
## 1 1 (1-1,1-1) arrange gtable[layout]
## 2 2 (1-1,2-2) arrange gtable[layout]
```

```
png(file = paste0(subDir,"/FIGURE_5_httk_moe_vs.png"), bg = "transparent",width=20,height=12,units="in",res=300)
print(grid.arrange(p4a,p4b, nrow = 1))
```

```
## TableGrob (1 x 2) "arrange": 2 grobs
##   z     cells    name           grob
## 1 1 (1-1,1-1) arrange gtable[layout]
## 2 2 (1-1,2-2) arrange gtable[layout]
```

```
dev.off()
```

```
## png 
##   2
```

Figure 5 Caption

```
HTTK.Toxtree$PREFERRED_NAME[which(HTTK.Toxtree$TTC_MoE > HTTK.Toxtree$NOAEL_MoE)]
```

```
## [1] "Perfluorooctanesulfonic acid" "Perfluorononanoic acid"
```

```
summary(nvo.reg)$r.squared #0.38
```

```
## [1] 0.3828382
```

```
summary(nvt.reg)$r.squared #0.59
```

```
## [1] 0.5902692
```

Median MoE Ratios

```
median(httk.overlap$NOAEL_MoE/httk.overlap$OED_MoE) #97.4
```

```
## [1] 97.42965
```

```
median(httk.overlap$NOAEL_MoE/httk.overlap$TTC_MoE) #2000
```

```
## [1] 2000
```

### Figure 6

```
cbPalette <- c("#E69F00", "#56B4E9","#009E73", "#CC79A7", "#D55E00", "#F0E442", "#0072B2")
#Setting a colorblind friendly palette
HTTK.Toxtree$oedlessexp <- HTTK.Toxtree$oed.AC50p5 / HTTK.Toxtree$Exposure.Total.Upper95
p <- ggplot(data=HTTK.Toxtree[c(1:10,101:110,201:210,301:310,401:410,501:510,601:610)]) + geom_point(aes(x=reorder(PREFERRED_NAME,oedlessexp),y=oed.AC50p5, colour=TTC_Class),size = 4, stat = "identity")
p <- p + geom_linerange(aes(x=reorder(PREFERRED_NAME,oedlessexp),ymin=oed.AC50p5,ymax=oed.AC50p50,colour=TTC_Class),size = 1.5, stat = "identity")
p <- p + geom_point(aes(x=reorder(PREFERRED_NAME,oedlessexp),y=Exposure.Total.Upper95),color='black',size = 3, stat = "identity")
p <- p + geom_linerange(aes(x=reorder(PREFERRED_NAME,oedlessexp),ymin=Exposure.Total.Median,ymax=Exposure.Total.Upper95),color='black',size = 1.5, stat = "identity") + scale_y_log10(labels = trans_format("log10", scales::math_format(10^.x)))
p <- p + geom_vline(xintercept = seq(10.5,60.5,10), linetype = 2)
p <- p  + scale_x_discrete(label=function(x) abbreviate(x, minlength=25)) +
  theme_bw()+
  scale_color_manual(values = cbPalette)+
  coord_flip()+
  theme(
    axis.text.x = element_text(size = 30, angle = 0,
                                   hjust = 0, vjust = 0, colour = "grey50", face = 'bold'),
        # axis.text.x = element_text(size = 25, angle = 90,
        #                            hjust = 1, vjust = 0.5, colour = "grey50", face = 'bold'),
        axis.ticks.x = element_line(size=0.01, color = 'grey50'),
        legend.title = element_blank(),
        legend.text = element_text(size=25, face = 'bold'),
        axis.title = element_text(size = 30, face = 'bold'),
        axis.text.y = element_text(size = 20, face = 'bold')) +
  labs(x='Compound',
       y='OED and Inferred Exposures \n (mg/kg/day)\n')

print(p)
```

```
png(file = paste0(subDir,"/FIGURE_6_ttc-layered_httk_aer_plot_oed_order_dots_reversed.png"), bg = "transparent",width=16,height=20,units="in",res=300)
print(p)
dev.off()
```

```
## png 
##   2
```

Finally, write the TTC files into the Risk Prioritization Folder

```
write.csv(select(HTTK.Toxtree,c('PREFERRED_NAME','CAS','QSAR_READY_SMILES','DTXSID','Structural Alert for genotoxic carcinogenicity','Structural Alert for S. typhimurium  mutagenicity','FG52_2','FG81_2','Cramer rules, with extensions','TTC_Class','Exposure.Total.Upper95','TTC_ug.kg.d','oed.AC50p5','NOAEL','TTC_MoE','OED_MoE','NOAEL_MoE')),
          paste0(subDir,"/HTTK_Output.csv"),row.names = F)
write.csv(select(CERAPP.Toxtree,c('preferred_name','casrn','SMILES','dsstox_substance_id','Structural Alert for genotoxic carcinogenicity','Structural Alert for S. typhimurium  mutagenicity','FG52_2','FG81_2','Cramer rules, with extensions','TTC_Class','Exposure.Total.Upper95','TTC_ug.kg.d','oed.AC50p5','NOAEL','TTC_MoE','OED_MoE','NOAEL_MoE')),
          paste0(subDir,"/CERAPP_Output.csv"),row.names = F)
write.csv(select(gHTTK.Toxtree,c('PREFERRED_NAME','CAS','QSAR_READY_SMILES','DTXSID','Structural Alert for genotoxic carcinogenicity','Structural Alert for S. typhimurium  mutagenicity','FG52_2','FG81_2','Cramer rules, with extensions','TTC_Class','Exposure.Total.Upper95','TTC_ug.kg.d','oed.AC50p5','NOAEL','TTC_MoE','OED_MoE','NOAEL_MoE')),
          paste0(subDir,"/HTTK_Output_genotox.csv"),row.names = F)
write.csv(select(gCERAPP.Toxtree,c('preferred_name','casrn','SMILES','dsstox_substance_id','Structural Alert for genotoxic carcinogenicity','Structural Alert for S. typhimurium  mutagenicity','FG52_2','FG81_2','Cramer rules, with extensions','TTC_Class','Exposure.Total.Upper95','TTC_ug.kg.d','oed.AC50p5','NOAEL','TTC_MoE','OED_MoE','NOAEL_MoE')),
          paste0(subDir,"/CERAPP_Output_genotox.csv"),row.names = F)
```
