# Supplementary figures and images for: Estimating provisional margins of exposure for data-poor chemicals using high-throughput computational methods
Source: Front Pharmacol. 2022 Oct 7;13:980747. doi: 10.3389/fphar.2022.980747 (PMC9586287; doi:10.3389/fphar.2022.980747)

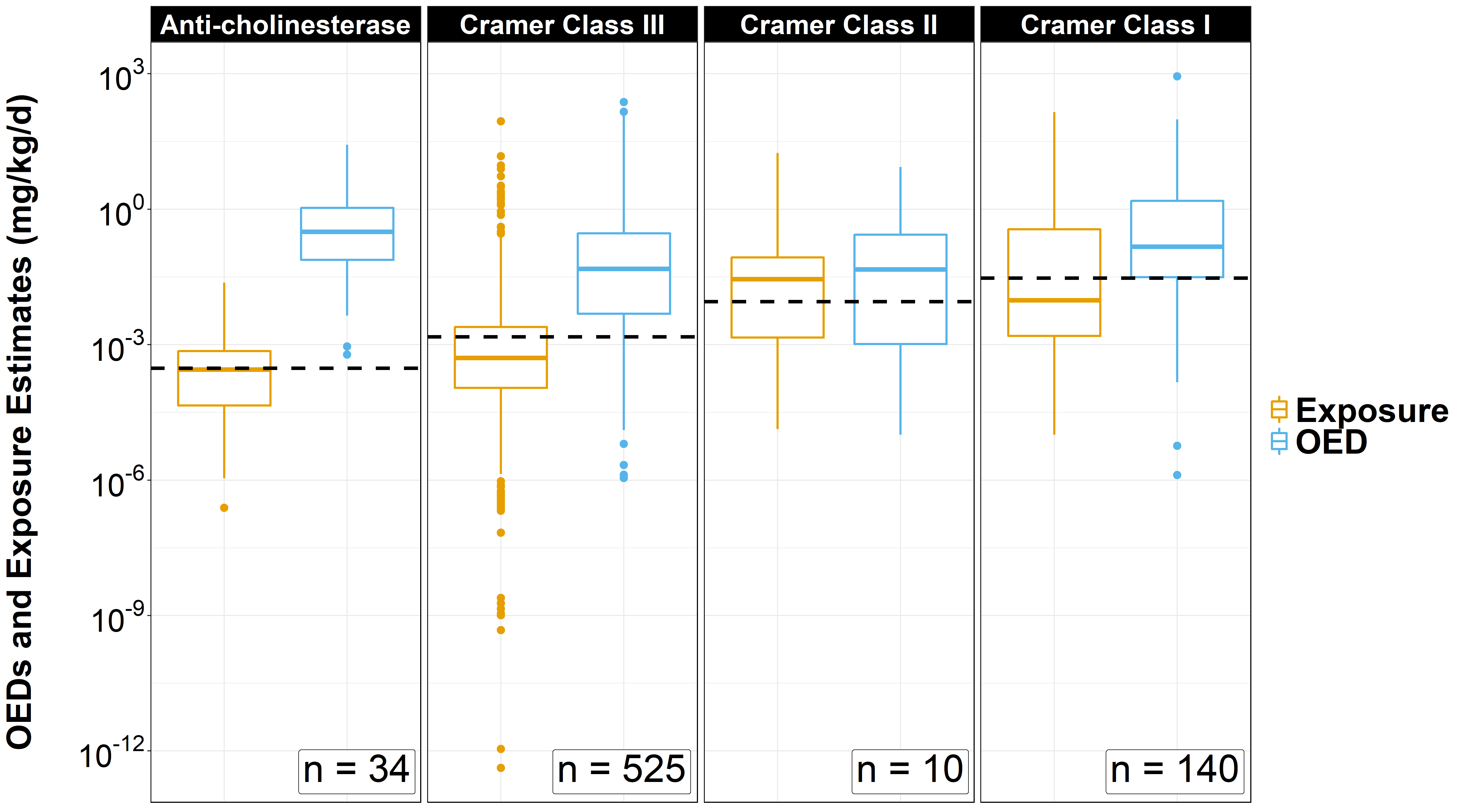

Supplement: Supplementary file 1 [file DataSheet1.ZIP › Supplemental_Files_full_submitted/Exposure_Case_Study/Risk_Prioritization/FIGURE_2_boxplot_httk_oedvseem_lines.png]

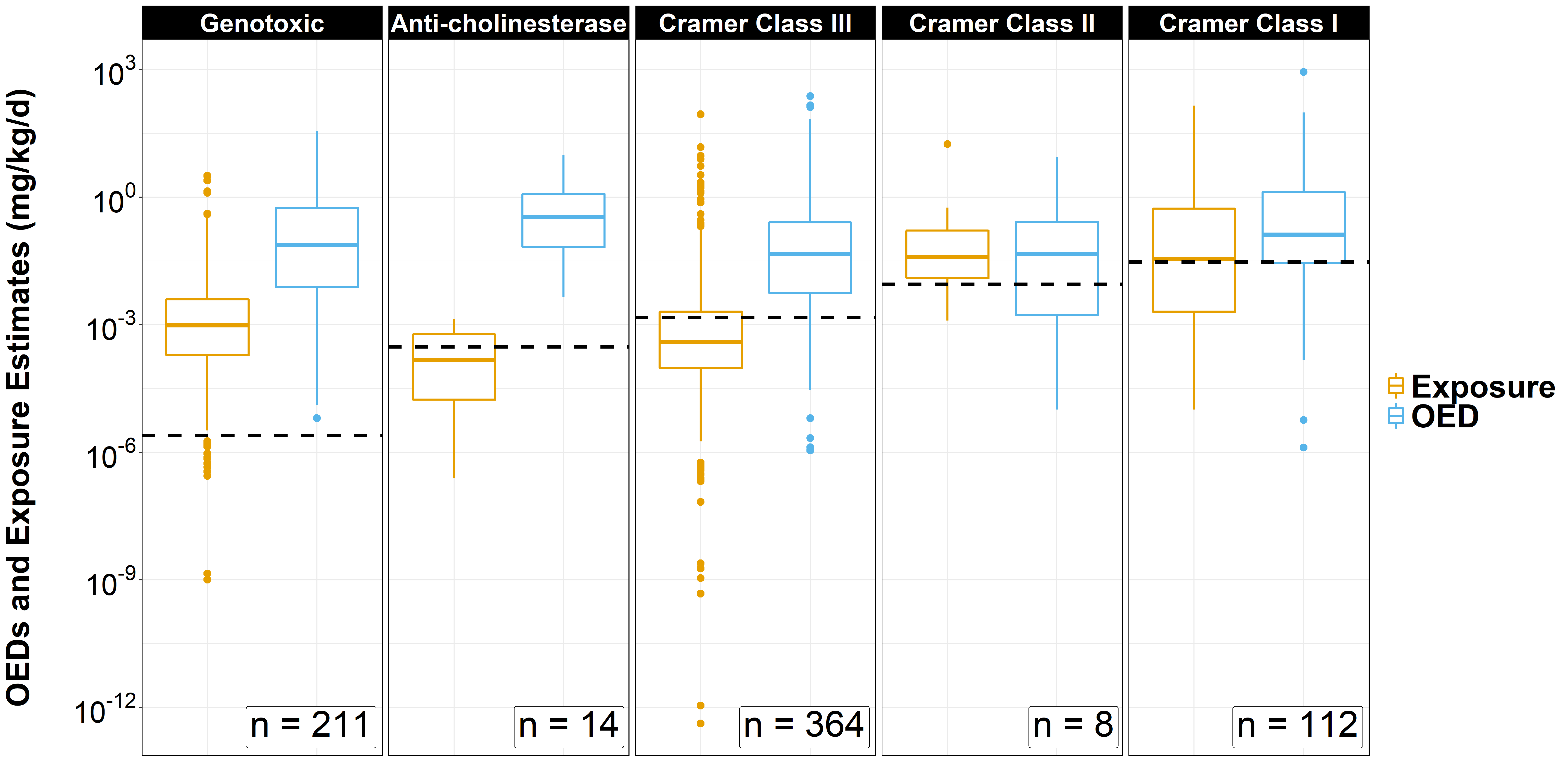

Supplement: Supplementary file 1 [file DataSheet1.ZIP › Supplemental_Files_full_submitted/Exposure_Case_Study/Risk_Prioritization/FIGURE_2b_boxplot_httk_oedvseem_lines_genotox.png]

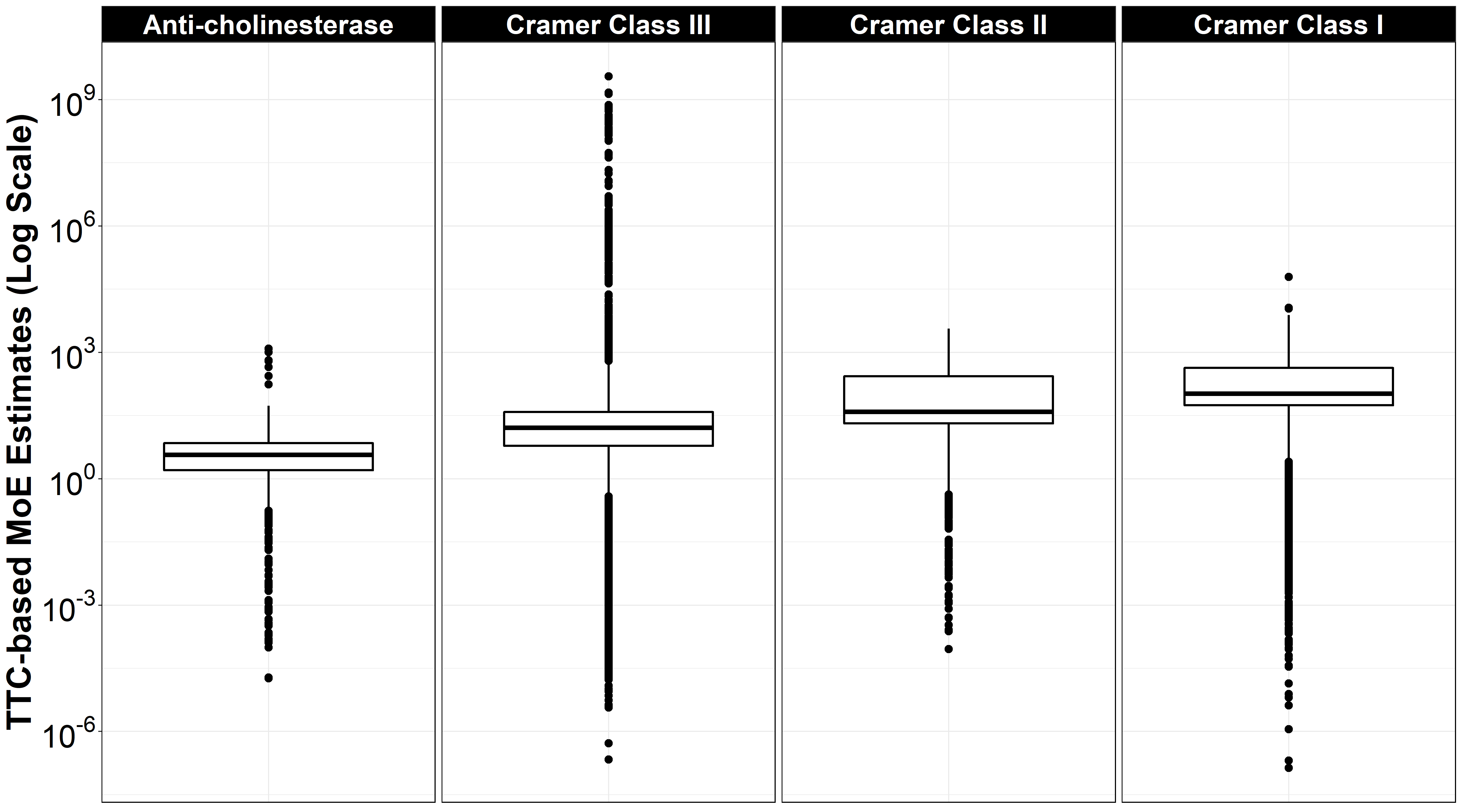

Supplement: Supplementary file 1 [file DataSheet1.ZIP › Supplemental_Files_full_submitted/Exposure_Case_Study/Risk_Prioritization/FIGURE_3_boxplot_cerapp_TTC_SEEM_MoE.png]

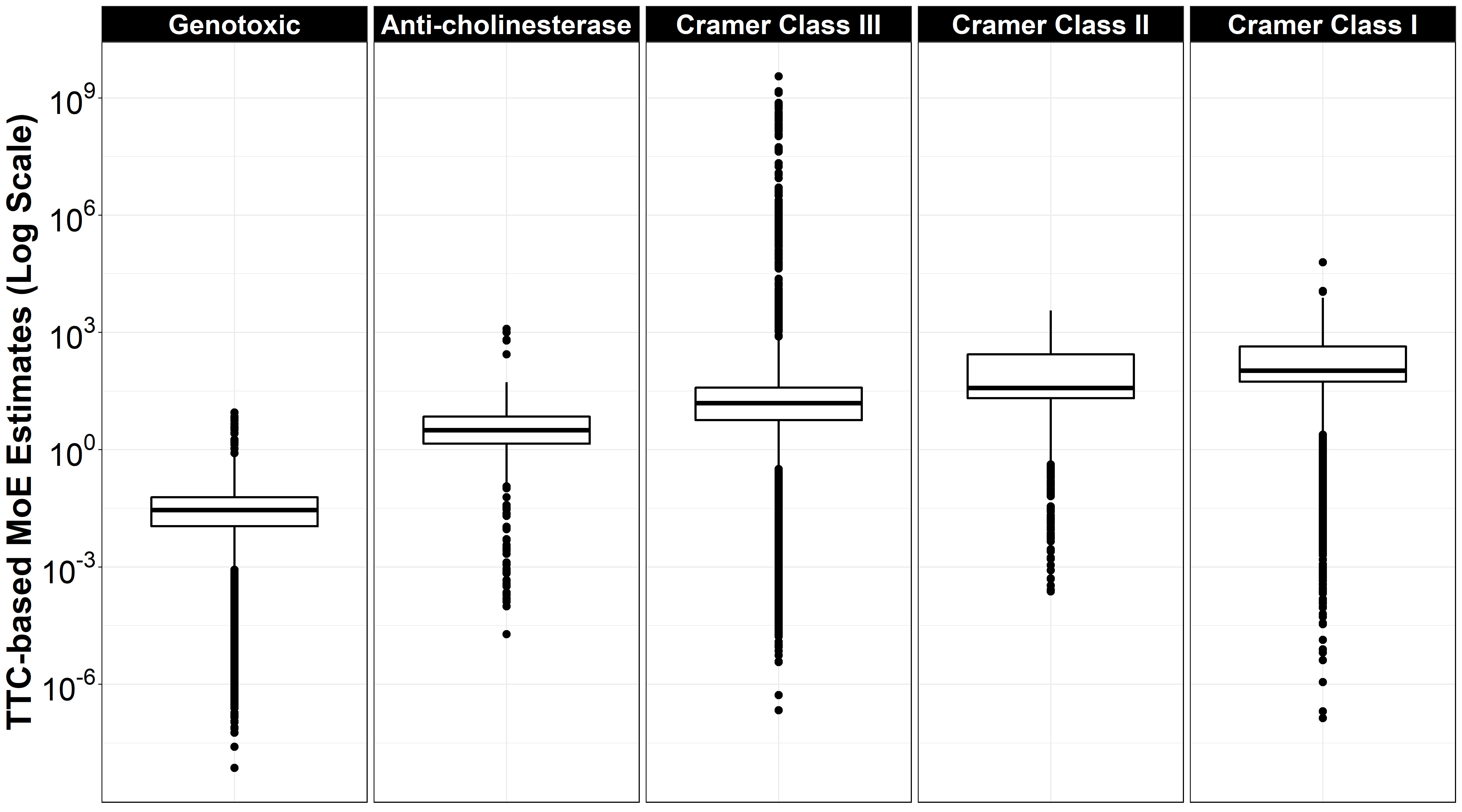

Supplement: Supplementary file 1 [file DataSheet1.ZIP › Supplemental_Files_full_submitted/Exposure_Case_Study/Risk_Prioritization/FIGURE_3b_boxplot_cerapp_TTC_SEEM_MoE_genotox.png]

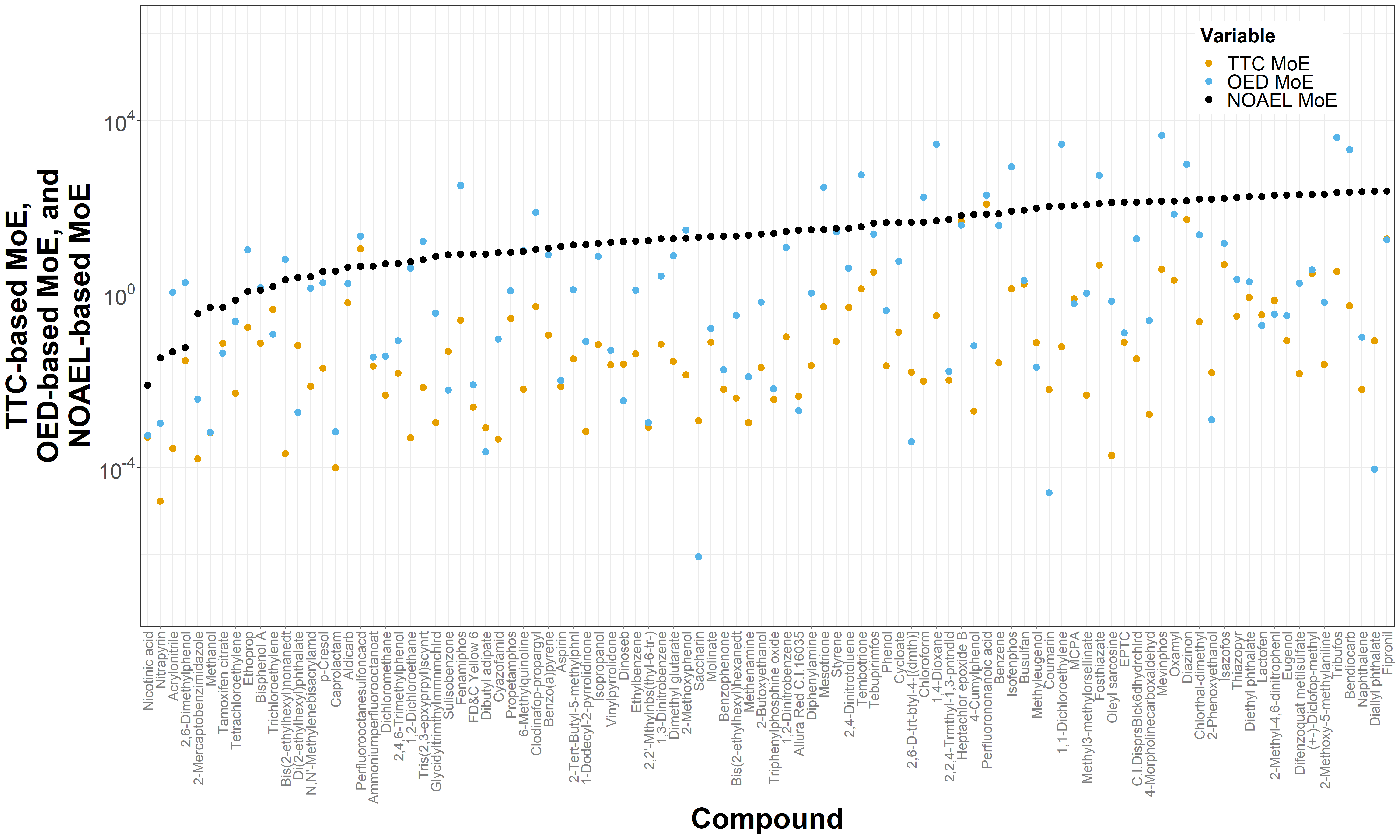

Supplement: Supplementary file 1 [file DataSheet1.ZIP › Supplemental_Files_full_submitted/Exposure_Case_Study/Risk_Prioritization/FIGURE_4_httk_moe_all.png]

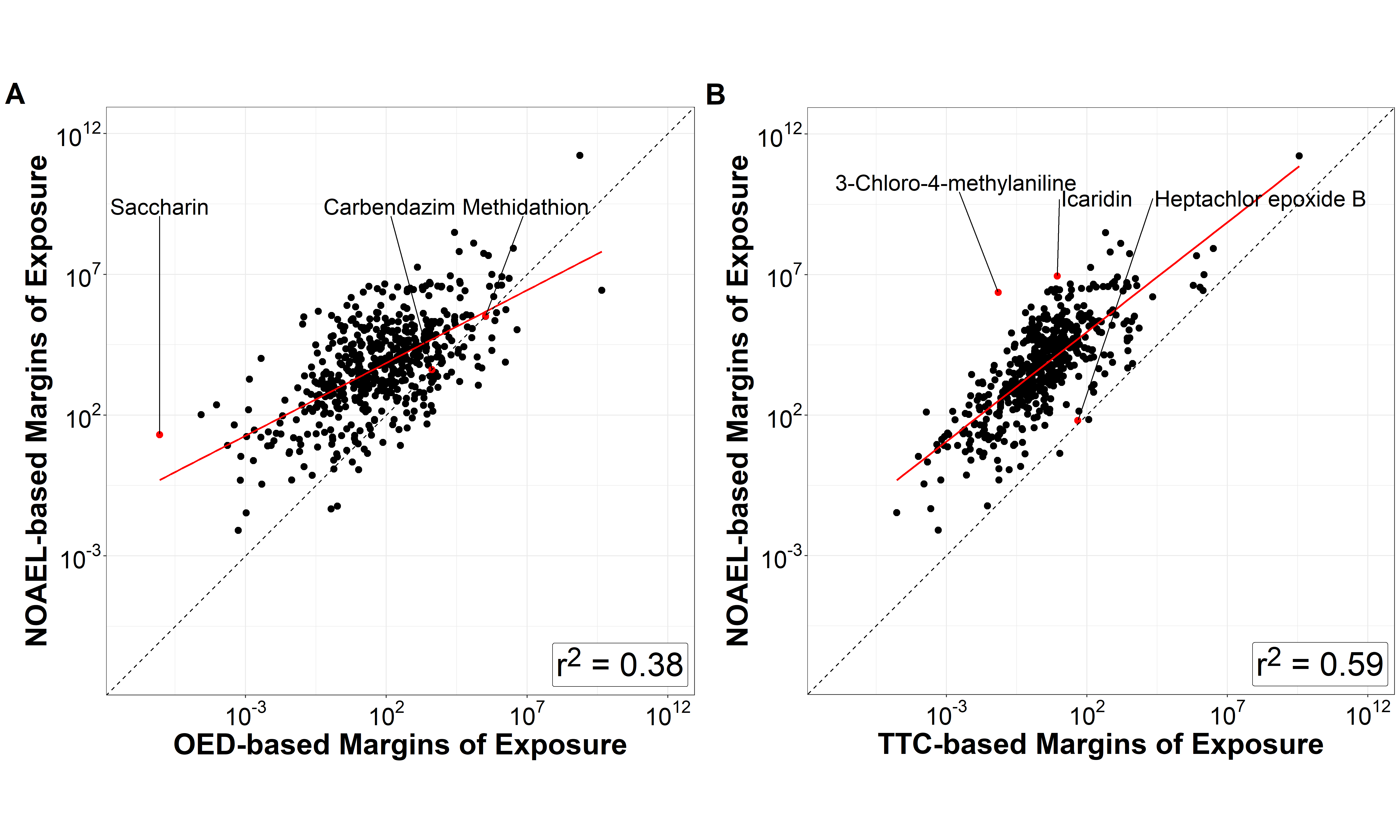

Supplement: Supplementary file 1 [file DataSheet1.ZIP › Supplemental_Files_full_submitted/Exposure_Case_Study/Risk_Prioritization/FIGURE_5_httk_moe_vs.png]

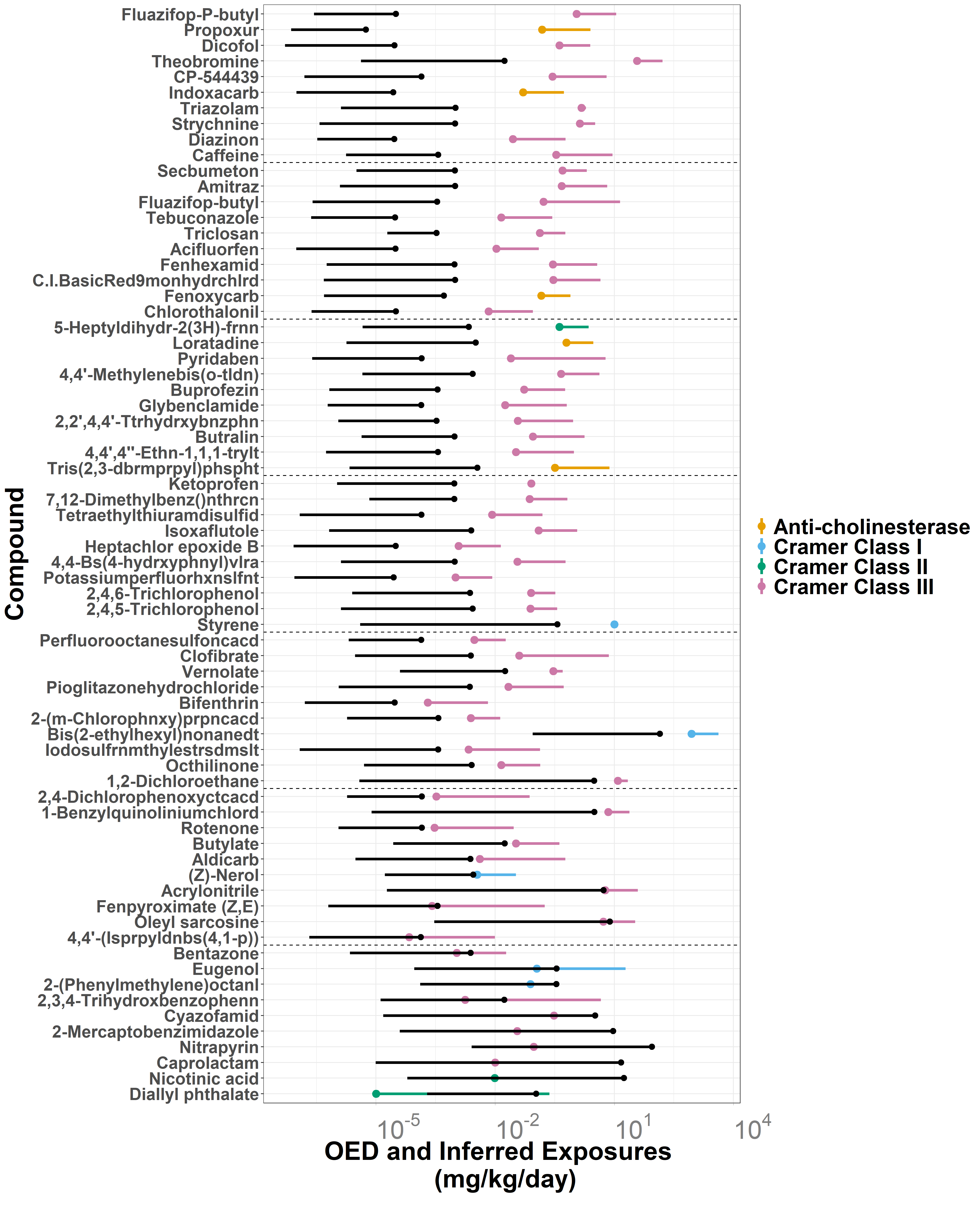

Supplement: Supplementary file 1 [file DataSheet1.ZIP › Supplemental_Files_full_submitted/Exposure_Case_Study/Risk_Prioritization/FIGURE_6_ttc-layered_httk_aer_plot_oed_order_dots_reversed.png]
